# Inflammatory response in bacteremia survivors and non-survivors: a case-control study

**DOI:** 10.1038/s41598-025-26183-x

**Published:** 2025-11-26

**Authors:** Markus JT Ojanen, Tapio Seiskari, Janne Aittoniemi, Heini Huhtala, Reetta Huttunen, Jaana Syrjänen, Ilkka Junttila, Marko Pesu, Juha Rannikko

**Affiliations:** 1 Faculty of Medicine and Health Technology, Tampere University, Tampere, Box 100, FI- 33014 Finland; 2 Department of Clinical Microbiology, Fimlab Laboratories, Arvo Ylpön katu 4, Tampere, FI- 33520 Finland; 3 Faculty of Social Sciences, Tampere University, Arvo Ylpön katu 34, Tampere, FI-33520 Finland; 4 Department of Internal Medicine, Tampere University Hospital, Box 2000, Tampere, FI- 33521 Finland; 5 Finnish Medicines Agency, Yliopistonkatu 38, Tampere, 33100 Finland; 6 Northern Finland Laboratory Center, Nordlab, Kiviharjuntie 11 B5, Oulu, 90220 Finland; 7 Research Unit of Biomedicine and Internal Medicine, University of Oulu, Aapistie 5A, Oulu, 90220 Finland

**Keywords:** Bacteremia, Sepsis, Biomarker, Cytokine, Cytokines, Biomarkers, Bacterial infection

## Abstract

Survival in bacteremia is influenced by host comorbidities, pathogen virulence, immune response, and the quality of medical care. A deeper understanding of host inflammatory responses may help identify new biomarkers and therapeutic targets to improve outcomes. In this study, we analyzed plasma samples from adult patients with bacteremia who presented to the emergency department. Patients who died within seven days (*n* = 44) were compared to matched survivors (*n* = 44) who lived at least 90 days, with matching based on age, sex, and causative bacterium. Relative concentrations of 92 inflammation-related plasma proteins were measured using the proximity extension assay (Olink Proteomics AB). Of these, 82 proteins were successfully quantified. Thirty proteins, including IL-20RA, CD40, and HGF, were significantly elevated in non-survivors, while three proteins—IFNG, Flt3L, and TNFB—were significantly reduced. Pathway enrichment analysis revealed activation of multiple proinflammatory signaling cascades in non-survivors, particularly those involving IL-6, IL-23, and IL-17. Logistic regression identified HGF, IL-17 C, and Flt3L as key discriminative proteins between groups. These findings indicate that the host inflammatory profile in bacteremia differs markedly between survivors and early non-survivors. Our results highlight potential diagnostic biomarkers for identifying high-risk patients and point to promising therapeutic targets in the management of bacteremia.

**Supplementary Information:**

The online version contains supplementary material available at 10.1038/s41598-025-26183-x.

## Introduction

While various risk factors for mortality in bacteremic patients have been described in the literature^1–3^, the pathobiology of severe infections remains largely unknown. In severe bacteremia cases, a common question is whether the bacteria were particularly virulent, the inflammatory response failed to function appropriately, or both^4^. It is known that certain bacteria, such as *Capnocytophaga canimorsus*, have higher rates of causing severe disease compared to, for instance, *Escherichia coli*^5^, and that underlying diseases such as metastatic cancer or hematological malignancies can disturb the normal host response and increase infection lethality^6,7^. Nevertheless, even patients with severe underlying conditions can survive of infection and *C. canimorsus* does not always cause severe disease. Therefore, more studies on both bacterial virulence and inflammatory response are still needed.

Many soluble macromolecules, including cytokines and eicosanoids, are known to play a role in the inflammatory response^8,9^. To better understand the interplay of these inflammatory mediators in the pathobiology of severe systemic infections, broad-scale assessment of their levels in different tissues is required. Relatively recent tools for identifying these mediators include assays capable of surveying dozens of different proteins from a single plasma sample^10^. These multiplex protein detection platforms can serve as screening tools for identifying proteins that warrant further investigation, e.g. as therapeutic targets in bacteremia.

In developed countries, patients who die within 30 days after the onset of bacteremia die mostly either because they have had a rapidly fatal underlying disease in which the bacteremia had been the last notch leading to death, or the death had been mostly attributable to the bacteremia itself^6,11^. Thereafter, deaths are primarily attributable to underlying diseases alone. In this study, patients who died within 7 days of bacteremia onset were selected as cases, while those who survived beyond 90 days were used as controls. Cases and controls were matched for sex, age, and bacterial species. We assessed the relative levels of 92 different proteins in plasma and analyzed which of these were associated with a lethal outcome.

## Methods

### Patient inclusion criteria and stratification

Tampere University Hospital is a tertiary hospital in Finland with a catchment population of approximately 530,000 inhabitants. Adult patients presenting to the hospital emergency department with a positive blood culture (excluding contaminants) were included in previous publications^6,12,13^. From this cohort of 481 cases with available plasma sample, all patients (*N* = 44) who died within 7 days after positive blood culture were selected as cases for this study. From the remaining original cohort, controls were selected based on survival beyond 90 days, matching the same bacterial species, sex, and closest age to the corresponding case.

Blood cultures were collected in BacT/Alert FA Plus (aerobic) and FN Plus (anaerobic) blood culture bottles and processed using the automated microbial detection system BacT/Alert 3D (bioMérieux, Marcy l’Etoile, France). All cases with *E. coli*,* Staphylococcus aureus*,* Streptococcus pneumoniae*,* Enterococcus faecalis*,* C. canimorsus*,* Pseudomonas aeruginosa*,* Haemophilus influenzae*, and *Klebsiella pneumoniae* bacteremia had controls with the same bacterial species. For cases with viridans group streptococci bacteremia, the controls did not necessarily have the same species within the viridans group. Some cases with anaerobic bacteremia had controls with a different species or subspecies within anaerobic bacteria. Controls for cases with polymicrobial bacteremia did not need to have the same bacteria but were also polymicrobial.

## Laboratory analysis

Plasma samples from the day of admission to the hospital were obtained generally at the same time as the blood culture, but with a maximum difference of 24 h, and were stored at − 80 °C. Both the plasma sample and blood culture were obtained a median of 37 min after arrival to the emergency department (interquartile range, 23–68 min). Antibiotic therapy was initiated later, except in 4 patients (4.5%) who received antibiotics before the plasma sample was collected. The expression levels of 92 proteins in plasma were determined using the proximity extension assay^10^ with the Olink Target 96 Inflammation panel by Olink Proteomics (Uppsala, Sweden). The values were normalized protein expression values, which are relative and not definitive concentrations. All clinical samples were analyzed on the same 96-well plate, with 8 wells reserved for internal and negative controls. The assay was outsourced to Biomedicum Functional Genomics Unit (Helsinki, Finland). The full names of the protein abbreviations can be found on the company website^14^ and in Supplementary Table 1. Proteins were included in the subsequent analyses if more than 93% of samples had detectable levels of the corresponding protein.

## Pathway analyses

Panther (v. 19.0)^15^ and Reactome PathwayBrowser (v. 3.7)^16^ were used to identify enriched biological processes and pathways in the differentially expressed proteins between case and control groups. Further curation of the enriched pathways was performed using InteractiVenn^17^.

### Statistical analysis

McNemar and Wilcoxon signed-rank tests were used to analyze the statistical difference between cases and controls. Logistic regressions were performed as conditional (fixed-effects) with forward method and were confirmed using the backward method. All missing data was handled by exclusion. To account for multiple comparisons, p-values were adjusted using the Benjamini–Hochberg False Discovery Rate (FDR) procedure. SPSS version 29.0 (IBM Corp., Armonk, NY, USA) and STATA version 18.5 (StataCorp LLC, Texas, USA) were used for statistical analyses.

### Ethics

The study was approved by the Ethics Committee of Tampere University Hospital, Finland (permit #R11099), and by the National Supervisory Authority for Welfare and Health. The requirement for informed consent was waived by the authorities, as no additional blood sampling was performed and routine patient care was not modified. The study was conducted in accordance with the Declaration of Helsinki.

## Results

**Fig. 1:**
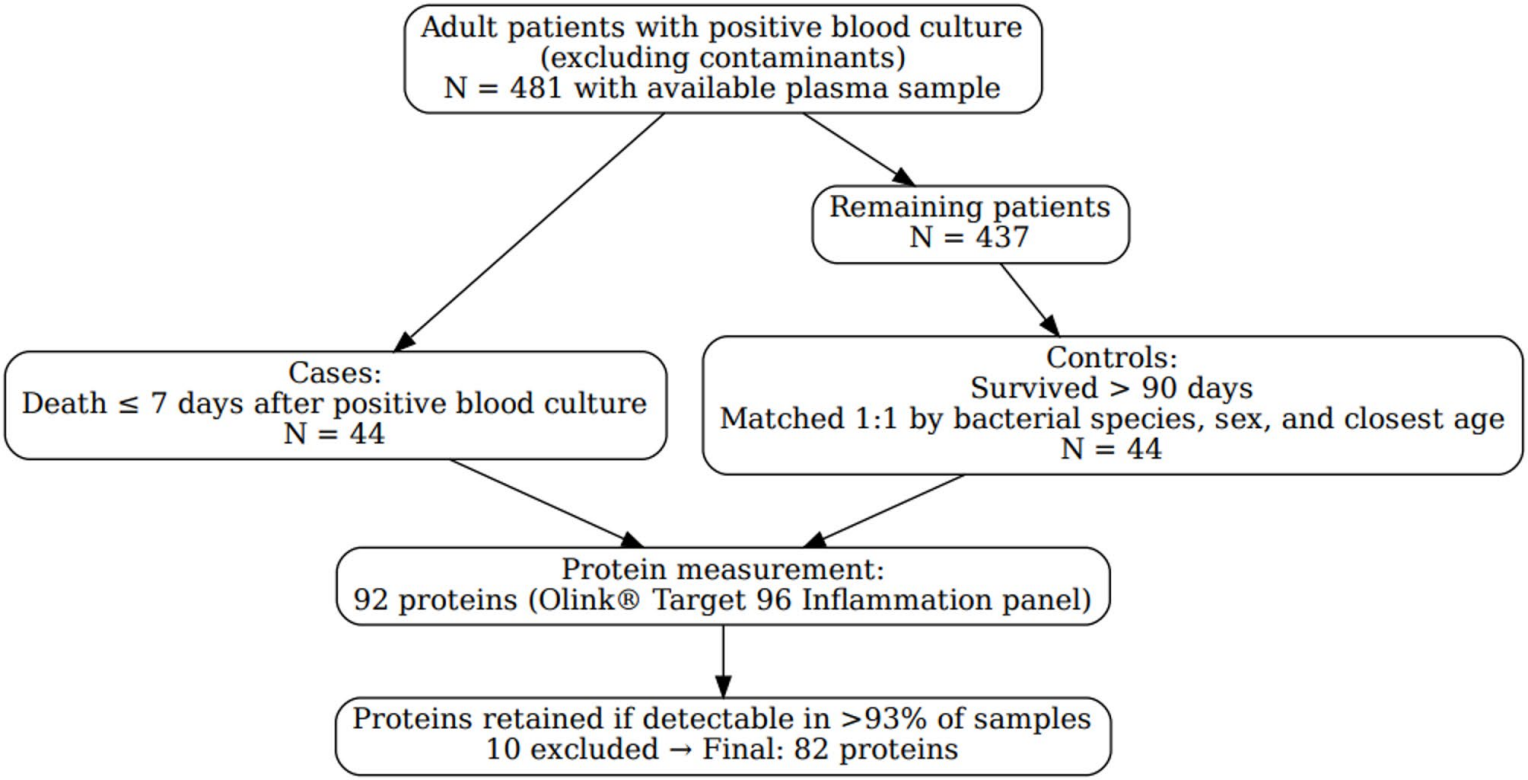
Flowchart illustrating patient selection, case-control allocation, and exclusions.

The 44 patients selected as cases in our study represented all patients who died within 7 days after bacteremia in our original bacteremia cohort of 481 patients^6^. A flowchart illustrating inclusions and exclusions is provided in Fig. 1.

The controls were stratified according to bacterial species, sex, and age and therefore showed no statistical difference between cases and controls (Table 1).

**Table 1:** Characteristics, underlying diseases, infection foci, causative organisms, and the severity of the disease in 84 bacteremia patients.

	Cases *N* = 42 *n* (%)	Controls *N* = 42 *n* (%)	All *N* = 84 *n* (%)	*P*-value
**Demographic**				
Median age, y (inter quartile range)	74 (66–79)	74 (65–81)	74 (12.1)	
Male	26 (62)	26 (62)	52 (62)	
**Underlying diseases**				
Heart disease	22 (52)	17 (40)	39 (46)	0.332
Diabetes mellitus, any type	15 (36)	16 (38)	31 (37)	1.000
Neurological	13 (31)	6 (14)	19 (23)	0.092
Liver disease	8 (19)	5 (12)	13 (15)	0.453
Solid tumor with metastasis	6 (14)	6 (14)	12 (14)	1.000
Alcohol abuse in the past 12 months	8 (19)	2 (4)	10 (12)	0.070
Hematological malignancy	7 (17)	1 (2)	8 (10)	0.070
No underlying diseases	1 (2)	4 (10)	5 (6)	0.375
**Infection focus**				
Unknown	15 (36)	15 (36)	30 (36)	1.000
Gastro-intestinal	11 (26)	12 (29)	23 (27)	1.000
Urinary	9 (21)	9 (21)	18 (21)	1.000
Lung	4 (10)	3 (7)	7 (8)	1.000
Other	3 (7)	3 (7)	6 (7)	1.000
**Causative organism**				
Gram+	11 (26)	11 (26)	22 (26)	
Gram-	20 (48)	20 (48)	40 (48)	
Anaerobic	4 (10)	4 (10)	8 (10)	
Polymicrobial	7 (17)	7 (17)	14 (17)	
**Severity**				
Day 0 C-reactive protein, median (quartiles)	136 (41–249)	95 (32–190)	110 (39–210)	0.069
Day 0 leucocyte count, median (quartiles). (Data on 41 controls.)	12.4 (6.0–21.6.0.6)	12.6 (8.5–16.8)	12.6 (6.7–17.9)	0.472
Septic shock	16 (38)	2 (5)	18 (21)	< 0.001*
Use of vasopressors	15 (36)	2 (5)	17 (18)	< 0.001*
Admitted from Emergency Department to Intensive Care Unit	13 (31)	2 (5)	15 (18)	0.001*

Additionally, there were no statistically significant differences in underlying diseases or infection foci. The severity of the disease (septic shock, use of vasopressors, and admission to the intensive care unit) was significantly worse in the cases than in the controls (*P* ≤ 0.001).

The protein panel’s internal quality control measurements raised a warning for one case-control pair and one control sample. The corresponding case sample from the latter was also excluded, resulting in the exclusion of two case and two control samples from the analyses. Out of the 92 proteins analyzed using the proximity extension assay, the levels of 82 proteins were further compared between the case and the control samples, whereas 10 proteins (Beta-NGF, IL-33, IL-13, IL-1 A, IL-2, IL-5, IL-4, IL-22RA1, TSLP, and IL-2RB) were excluded from the analyses due to a high number of samples falling below the limit of detection for the corresponding protein.

**Fig. 2:**
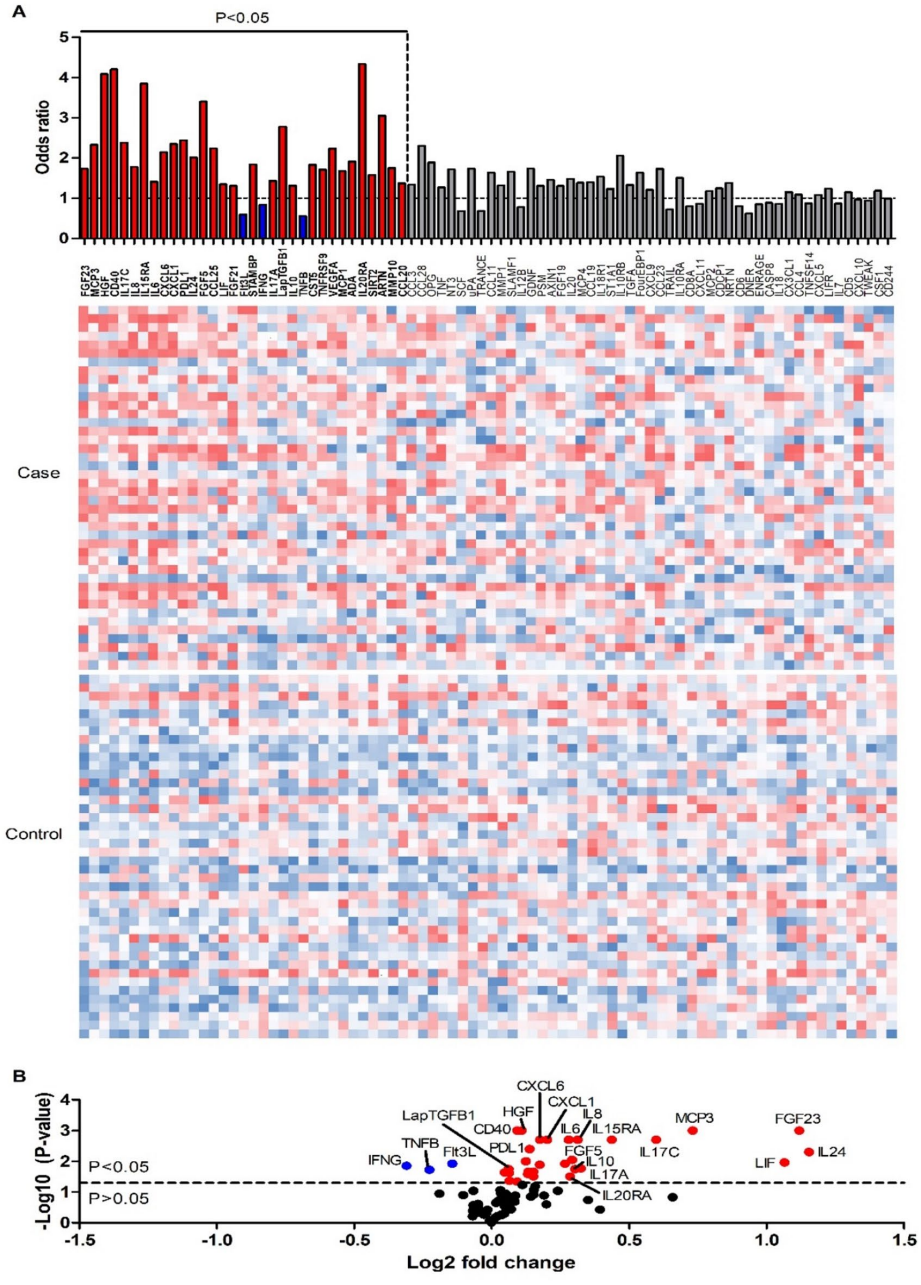
Bacteremia cases have an altered inflammatory response in bacteremia. (**A**) Heatmap of protein expression levels in 84 bacteremia cases, ordered by statistical significance. Columns representing proteins with significantly higher odds ratios in cases compared to controls are marked in red, while those with significantly lower odds ratios are marked in blue. Proteins without statistically significant differences are marked in grey. Red indicates high relative expression, and blue indicates low relative expression for each protein. Color scales have been normalized separately for each protein, with the range adjusted between the lowest and highest expression values. (**B**) Volcano plot representing the p-values (–log10 scale) and average fold changes (log2 scale) of protein expression levels. Upregulated proteins (red) with the lowest p-values, highest fold changes, and highest odds ratios, as well as all downregulated proteins (blue), are indicated in the figure. The dotted line represents the p-value threshold of 0.05 (approximately 1.30). Note that ARTN is not included in the graph due to a negative average expression value in the control group, which results in a complex number for the log2 fold change.

Figure 2 shows the odds ratios, the relative protein levels and the average fold changes together with the P-values, whereas Supplementary Table 2 shows missing relative values, medians, and inter quartile ranges.

Of the 82 proteins, 33 had statistically significant odds ratios (ORs) between cases and controls. In majority of these (*n* = 30), cases had higher expression level than controls (OR > 1). In other words, the high levels of these 30 proteins in plasma were associated with death within 7 days. IL-20RA, CD40, HGF, IL-15RA, FGF-5, and ARTN showed the highest ORs (4.34, 4.21, 4.08, 3.85, 3.40, 3.05, respectively). Conversely, lower abundance of IFNG, Flt3L and TNFB (OR 0.84, 0.59, and 0.56, respectively) were observed in the case group, indicating that reduced levels of these proteins in plasma are associated with poor prognosis in patients with bacteremia. All ORs, 95% confidence interval (CIs), and p-values are shown in Supplementary Table 3. The Benjamini–Hochberg False Discovery Rate (FDR) was also tested (Supplementary Table 4). An FDR threshold of 0.12 corresponded to the same biomarker selection as a p-value threshold of 0.05 in this study.

To explore the signaling pathways associated with prognosis, we next analyzed the differentially expressed proteins between cases and controls (30 up-regulated and 3 down-regulated) using the Panther overrepresentation test^15^. To exclude possible selection bias caused by the pre-selected protein panel (Olink Target 96 Inflammation panel), we also analyzed the enriched pathways in the background list of proteins (49 analytes) that showed no differences in the expression levels between cases and controls. Additionally, analysis was performed to all proteins (82 analytes) for comparison. We identified 253 biological processes that were enriched within the upregulated list of proteins (Supplementary Fig. 1, Supplementary Table 5). Specifically, we identified several pro-inflammatory cytokine signaling pathways, such as “regulation of interleukin-6 production” (Gene Ontology ID (GO):0045408, *P* = 2.69 × 10^− 6^), “regulation of interleukin-23 production” (GO:0045396, *P* = 9.18 × 10^− 5^) and “positive regulation of interleukin-17 production” (GO:0032740, *P* = 7.05 × 10^− 4^). The downregulated proteins (IFNG, Flt3L, TNFB) did not reveal statistically significant pathway enrichment. We also verified the pathways obtained with the upregulated proteins using Reactome PathwayBrowser (v. 3.7.) (Supplementary Table 6). While the analysis identified some previously described pathways in the target group (e.g. “Interleukin-6 family signaling”), background group (e.g. “Interleukin-1 family signaling”) and target/background group (e.g. “Interleukin-10 signaling”), certain pathways such as “regulation of IL-17 production” and “regulation of IL-23 production” were not found, indicating that the pathway analyses do not have complete overlap.

To identify the key analytes within the groups (highest/lowest ORs, IL-17/Th17 cytokines), we next performed logistic regression analyses. In the proteins with highest odds ratio HGF remained statistically significant (OR 4.11, 95% CI 1.72–9.79, *P* = 0.001). When the logistic regression was done on the differentially expressed IL-17/Th17 associated proteins (IL-17 C, IL-17 A, LAPTGFbeta1, IL-8, IL-6, and CXCL-1), only IL-17 C remained statistically significant (OR 2.38, 95% CI 1.38–4.11, *P* = 0.002). Finally, in the logistic regression analysis of the downregulated proteins, only Flt3L remained statistically significant (OR 0.59, 95% CI 0.39–0.89, *P* = 0.012).

## Discussion

To analyze the differences in the inflammatory response between bacteremia survivors and non-survivors, we quantified the relative levels of 92 proteins using a commercially available proximity extension assay platform (Olink). Of these, 82 proteins were reliably quantifiable, and 33 showed statistically significant differences between cases and controls. Further analysis of the differentially expressed proteins using overrepresentation tests revealed their involvement in several biological processes. More specifically, the analysis suggested that non-survivors exhibited alterations in various aspects of the immune response, including B cell activation, immunoglobulin production, and IL-17 family cytokine signaling, with several associated GO terms reflecting these processes. However, reflecting the complexity of the immune response/biological processes occurring in bacteremia patients, not only pro-inflammatory- but also anti-inflammatory- pathways e.g. interleukin-10 signaling were enriched within the differentially expressed proteins. It is additionally noteworthy that analyzing the background list of proteins (no significant difference between cases and controls) also revealed many immunological processes, such as “interleukin-10 mediated signaling pathways” (GO: 0140105, *P* = 3.76 × 10^− 4^) and “positive regulation of canonical NF-kappaB signal transduction” (GO:0043122, *P* = 7.89 × 10^− 7^), which highlights the importance of additional tools to mitigate possible biases caused by pre-selected proteins in pathway analysis. In fact, although there were some similarities between the two pathway analysis platforms used in the current study, not all pathways identified with Panther were also identified using Reactome, and vice versa. Collectively, although care should be taken in drawing conclusions from the overrepresentation data, the broad-scale assessment of plasma protein levels enables the identification of key biological processes involved in severe systemic infections.

Logistic regression of the proteins with odds ratio > 3 (IL-20RA, CD40, HGF, IL-15RA, FGF-5, and ARTN) indicated that Hepatocyte Growth Factor (HGF) was the only one that remained statistically significant. HGF is a multifunctional cytokine with roles in angiogenesis, tumorigenesis, and tissue regeneration, among other processes^18^. Beyond its relevance in various other fields of study, this cytokine has also been investigated in the context of infection. Nayeri et al. reported that serum HGF levels were significantly higher in patients with acute infectious diseases compared to healthy controls^19^. Similarly, Sekine et al. found that high plasma HGF levels were significantly correlated with the presence of infection^20^. Furthermore, Peng et al. demonstrated that elevated HGF levels in sepsis patients were indicative of poor prognosis^21^, which aligns with our current findings in bacteremia patients.

Fms-related tyrosine kinase 3 ligand (Flt3L), interferon gamma (IFNG), and lymphotoxin-alpha/tumor necrosis factor-beta (TNFB) had statistically significantly lower odds ratio among cases compared to controls. In logistic regression analysis, only Flt3L remained statistically significant. This cytokine and growth factor increases the number of immune cells, particularly by serving as a progenitor for dendritic cells^22^. Experimental studies have demonstrated potential therapeutic effects of Flt3L; for example, treatment with Flt3L increased survival in mice subjected to a subsequent burn wound infection^23^. Flt3L is generally upregulated in response to infection^24,25^. Thus, patients with normal or elevated levels appear to exhibit a better-balanced and more effective cellular immune response against the causative agent.

Logistic regression analysis also identified IL-17 C expression as significantly different between cases and controls. IL-17 C is an epithelial-derived cytokine, and its expression is regulated by both Toll-like receptors and cytokines, including IL-1B, TNF, and IL-17 A^26^. Thus, IL-17 C expression may be induced by both bacterial components and the host immune response. Functionally, IL-17 C plays a dual role by regulating the expression of antimicrobial peptides on epithelial surfaces^27^ and promoting the expression of pro-inflammatory cytokines, including IL-1B, TNF, and IL-6^26^. This dual role suggests that IL-17 C might be particularly interesting candidate for further studies for sepsis survival, as its expression may be regulated by both pathogen structures and host immune mechanisms.

There are a few limitations to this study that could be addressed. In our study, 36% of patients had an unknown focus of infection. This proportion is slightly higher than in other bacteremia studies, even though those also included patients outside intensive care units^28^. The likely reason is that half of the patients (all cases) died within 7 days, which has been shown to increase the proportion of unknown foci^29^. Cases and controls did not differ significantly in the prevalence of underlying diseases. However, alcohol abuse, hematological malignancies, and neurological diseases were several times more common in cases than in controls. We believe that in a larger cohort, these differences might have reached statistical significance. Additionally, while we matched cases and controls by bacterial species, they did not originate from the same strain. In the case of viridans streptococci, controls belonged to the same genus but not necessarily the same species. Our analysis was limited to 92 proteins rather than the entire proteome, which limits the resolution and can cause selection bias in overrepresentation analyses. Furthermore, if HGF, IL-17 C, and Flt3L are to be used as diagnostic analytes, absolute quantification with pre-determined cut-offs would be required, instead of relative quantities.

Much research remains to be done to understand the pathogenesis of bacteremia. In our study, we identified 33 proteins with significantly different plasma levels at admission between surviving and non-surviving bacteremia patients. Among these, HGF, IL-17 C, and Flt3L remained significant in logistic regression. Combined with previous findings, our results suggest that these proteins warrant further investigation in the field of infectious diseases. Future studies could include, for instance, inflammatory mediator knockout mouse models or the infusion of Flt3L in animal models of bacteremia. Ultimately, such research may lead to the identification of novel therapeutic targets to prevent infections from worsening the patient’s condition.

## Supplementary Information

Below is the link to the electronic supplementary material.

Supplementary Material 1

## Data Availability

Upon reasonable request from the corresponding author.
