# Supplementary material for: Inflammatory response in bacteremia survivors and non-survivors: a case-control study
Source: Sci Rep. 2025 Nov 26;15:42134. doi: 10.1038/s41598-025-26183-x (PMC12657952; doi:10.1038/s41598-025-26183-x)
Supplement: Supplementary file 1 — Supplementary Material 1 [file 41598_2025_26183_MOESM1_ESM.pdf]

Supplementary Tables 1-6 and Figure 1

Inflammatory Response in Bacteremia Survivors and Non-Survivors: A Case-Control Study

Markus JT Ojanen[a] PhD, Tapio Seiskari[b] MD PhD, Janne Aittoniemi[b] MD PhD, Heini Huhtala[c] MSc, Reetta Huttunen[a,d]MD PhD, Jaana Syrjänen[a,d] MD PhD, Ilkka Junttila[a,b,f,g] MD PhD, Marko Pesu[a,e] MD PhD, and Juha Rannikko[a,d*] MD PhD

*Corresponding author, ORCID ID 0000-0002-7199-6306, e-mail: juha.rannikko@gmail.com

[a]Faculty of Medicine and Health Technology, Tampere University, Box 100, FI-33014 Tampere University, Finland

[b]Department of Clinical Microbiology, Fimlab Laboratories, Arvo Ylpön katu 4, FI-33520 Tampere, Finland

[c]Faculty of Social Sciences, Tampere University, Arvo Ylpön katu 34, FI-33520 Tampere, Finland

[d]Department of Internal Medicine, Tampere University Hospital, Box 2000, FI-33521 Tampere, Finland

[e]Finnish Medicines Agency, Yliopistonkatu 38, 33100 Tampere, Finland

[f]Northern Finland Laboratory Center, Nordlab, Kiviharjuntie 11 B5, 90220, Oulu, Finland.

[g]Research Unit of Biomedicine and Internal Medicine, Aapistie 5A, University of Oulu, 90220 Oulu, Finland.

Supplementary Table 1. The names and abbreviations of Olink® Target® 96 proximity extension analysis inflammation panel proteins

Protein name	Abbreviation
T-cell differentiation antigen CD6	CD6
Kit ligand	KITLG (MGF, SCF)
Interleukin-18	IL18 (IGIF, IL1F4)
Signaling lymphocytic activation molecule	SLAMF1 (SLAM)
Protransforming growth factor alpha	TGFA
C-C motif chemokine 13	CCL13 (MCP4, NCC1, SCYA13)
Eotaxin	CCL11 (SCYA11)
Tumor necrosis factor ligand superfamily member 14	TNFSF14 (HVEML, LIGHT)
Fibroblast growth factor 23	FGF23 (HYPF)
Interleukin-10 receptor subunit alpha	IL10RA (IL10R)
C-C motif chemokine 4	CCL4 (LAG1, MIP1B, SCYA4)
Fibroblast growth factor 5	FGF5
Leukemia inhibitory factor receptor	LIFR
Fibroblast growth factor 21	FGF21
C-C motif chemokine 19	CCL19 (ELC, MIP3B, SCYA19)
Interleukin-15 receptor subunit alpha	IL15RA
Interleukin-10 receptor subunit beta	IL10RB (CRFB4, D21S58, D21S66)
Interleukin-22 receptor subunit alpha-1	IL22RA1 (IL22R)
Interleukin-18 receptor 1	IL18R1 (IL1RRP)
Programmed cell death 1 ligand 1	CD274 (B7H1, PDCD1L1, PDCD1LG1, PDL1)
Beta-nerve growth factor	NGF (NGFB)
C-X-C motif chemokine 5	CXCL5 (ENA78, SCYB5)
Interstitial collagenase	MMP1 (CLG)
Tumor necrosis factor ligand superfamily member 11	TNFSF11 (OPGL, RANKL, TRANCE)
Thymic stromal lymphopoietin	TSLP
Interleukin-2	IL2
Vascular endothelial growth factor A	VEGFA (VEGF)
C-C motif chemokine 7	CCL7 (MCP3, SCYA6, SCYA7)
Glial cell line-derived neurotrophic factor	GDNF
CUB domain-containing protein 1	CDCP1 (TRASK)
Natural killer cell receptor 2B4	CD244 (2B4)
Interleukin-7	IL7
Tumor necrosis factor receptor superfamily member 11B	TNFRSF11B (OCIF, OPG)
Transforming growth factor beta-1 proprotein	TGFB1 (TGFB)
Urokinase-type plasminogen activator	PLAU
Interleukin-6	IL6 (IFNB2)
Growth-regulated alpha protein	CXCL1 (GRO, GRO1, GROA, MGSA, SCYB1)
Interleukin-17C	IL17C
Interleukin-17A	IL17A (CTLA8, IL17)
C-X-C motif chemokine 11	CXCL11 (ITAC, SCYB11, SCYB9B)
Axin-1	AXIN1 (AXIN)
Tumor necrosis factor ligand superfamily member 10	TNFSF10 (APO2L, TRAIL)
Interleukin-20 receptor subunit alpha	IL20RA
C-X-C motif chemokine 9	CXCL9 (CMK, MIG, SCYB9)
Cystatin-D	CST5
Interleukin-2 receptor subunit beta	IL2RB (IL15RB)
Interleukin-1 alpha	IL1A (IL1F1)
Oncostatin-M	OSM

C-C motif chemokine 2	CCL2 (MCP1, SCYA2)
Interleukin-8	CXCL8 (IL8)
Delta and Notch-like epidermal growth factor-related receptor	DNER (BET)
C-C motif chemokine 28	CCL28 (SCYA28)
NAD-dependent protein deacetylase sirtuin-2	SIRT2 (SIR2L, SIR2L2)
Interleukin-20	IL20 (ZCYTO10)
Eukaryotic translation initiation factor 4E-binding protein 1	EIF4EBP1
C-X-C motif chemokine 10	CXCL10 (INP10, SCYB10)
C-X-C motif chemokine 6	CXCL6 (GCP2, SCYB6)
Fms-related tyrosine kinase 3 ligand	FLT3LG
Protein S100-A12	S100A12
C-C motif chemokine 3	CCL3 (G0S19-1, MIP1A, SCYA3)
C-C motif chemokine 23	CCL23 (MIP3, MPIF1, SCYA23)
Tumor necrosis factor	TNF (TNFA, TNFSF2)
Interleukin-10	IL10
Stromelysin-2	MMP10 (STMY2)
Artemin	ARTN (EVN)
Interleukin-13	IL13 (NC30)
Interleukin-24	IL24 (MDA7, ST16)
Interleukin-12 subunit beta	IL12B (NKSF2)
T-cell surface glycoprotein CD5	CD5 (LEU1)
Hepatocyte growth factor	HGF (HPTA)
Tumor necrosis factor receptor superfamily member 5	CD40 (TNFRSF5)
Interferon gamma	IFNG
Macrophage colony-stimulating factor 1	CSF1
Lymphotoxin-alpha	LTA (TNFB, TNFSF1)
Adenosine deaminase	ADA (ADA1)
Interleukin-5	IL5
STAM-binding protein	STAMBP (AMSH)
Sulfotransferase 1A1	SULT1A1 (STP, STP1)
C-C motif chemokine 20	CCL20 (LARC, MIP3A, SCYA20)
Tumor necrosis factor ligand superfamily member 12	TNFSF12 (APO3L, DR3LG)
Interleukin-33	IL33 (C9orf26, IL1F11, NFHEV)
Neurotrophin-3	NTF3
Fractalkine	CX3CL1 (FKN, NTT, SCYD1)
C-C motif chemokine 25	CCL25 (SCYA25, TECK)
Caspase-8	CASP8 (MCH5)
C-C motif chemokine 8	CCL8 (MCP2, SCYA10, SCYA8)
Neurturin	NRTN
Leukemia inhibitory factor	LIF (HILDA)
Interleukin-4	IL4
Fibroblast growth factor 19	FGF19
Tumor necrosis factor receptor superfamily member 9	TNFRSF9 (CD137, ILA)
T-cell surface glycoprotein CD8 alpha chain	CD8A (MAL)

Supplementary Table 2. Number of measured biomarkers, averages, medians, and inter quartile ranges. The values are normalized protein expression (NPX) values (relative, not a definitive concentration).

Biomarker	Number measured	Number excluded	Average			Median			Inter Quartile Range					
			Case	*Control*	*All*	*Case*	*Control*	*All*	Case Q1	Case Q3	Control Q1	Control Q3	All Q1	All Q3
**FGF23**	84	0	6,38	2,94	4,66	7,65	2,66	4,97	4,98	8,24	0,71	4,84	2,05	7,72
**IL24**	78	6	3,54	1,66	2,63	3,30	1,40	2,22	2,04	4,86	0,86	2,22	1,19	3,53
**MCP3**	84	0	4,95	2,98	3,96	4,29	2,56	3,31	3,57	6,58	2,15	3,14	2,38	4,95
**IL17C**	84	0	5,74	3,79	4,76	5,62	3,63	4,48	4,34	7,49	3,04	4,49	3,29	5,84
**HGF**	84	0	11,76	10,90	11,33	12,09	10,92	11,30	11,23	12,36	10,39	11,34	10,65	12,13
**CD40**	84	0	12,68	11,89	12,29	12,72	11,73	12,37	12,24	13,29	11,22	12,74	11,56	12,97
**IL8**	84	0	9,92	7,99	8,95	10,27	7,60	8,43	8,04	11,93	6,75	8,78	7,09	11,34
**IL15RA**	84	0	2,70	1,99	2,34	2,51	1,71	2,26	2,15	3,52	1,34	2,63	1,51	3,10
**IL6**	84	0	11,25	9,26	10,25	12,25	9,02	10,01	9,40	13,41	7,33	11,03	8,18	13,14
**CXCL6**	84	0	9,52	8,43	8,97	9,38	8,33	8,71	8,33	10,43	7,88	9,01	8,04	9,79
**PDL1**	84	0	7,18	6,52	6,85	7,11	6,29	6,68	6,53	7,86	5,87	7,13	6,07	7,60
**CXCL1**	84	0	10,57	9,19	9,88	9,83	8,89	9,24	9,17	12,49	8,37	9,73	8,53	11,16
**LIF**	84	0	4,37	2,09	3,23	2,97	1,31	2,21	1,27	6,76	0,46	2,78	0,68	4,05
**IL20**	83	1	1,13	0,74	0,94	0,85	0,24	0,41	0,31	1,45	0,16	0,67	0,18	1,40
**CCL25**	84	0	6,93	6,36	6,64	7,12	6,38	6,71	6,55	7,50	5,59	7,15	5,90	7,36
**FGF5**	84	0	1,98	1,62	1,80	2,00	1,52	1,69	1,51	2,38	1,25	1,83	1,38	2,32
**FGF21**	84	0	9,06	7,54	8,30	9,32	7,03	8,38	7,62	10,95	6,08	9,31	6,71	10,08
**CST5**	84	0	5,78	5,20	5,49	5,55	4,98	5,22	5,04	6,28	4,61	5,66	4,81	6,16
**ARTN**	79	5	0,07	-0,34	-0,13	-0,14	-0,43	-0,27	-0,36	0,35	-0,58	-0,16	-0,54	0,10
**VEGFA**	84	0	12,44	12,04	12,24	12,61	12,05	12,19	11,93	13,04	11,72	12,34	11,80	12,78
**TNFRSF9**	84	0	7,85	7,16	7,50	7,70	7,15	7,38	6,76	8,91	6,36	7,68	6,43	8,49
**LAPTGFbeat1**	84	0	8,36	8,01	8,19	8,33	7,94	8,11	7,93	8,69	7,53	8,33	7,73	8,56
**IL10**	84	0	9,11	7,38	8,24	9,06	6,70	8,08	6,34	10,99	4,91	10,06	5,79	10,41
**MCP1**	84	0	13,70	13,11	13,40	14,05	12,99	13,52	13,02	14,59	12,25	13,92	12,39	14,32
**STAMBP**	84	0	5,65	5,01	5,33	5,61	4,86	5,08	4,54	6,56	4,49	5,41	4,53	6,02
**IL17A**	83	0	5,29	4,23	4,76	5,29	4,41	4,76	3,31	7,06	3,02	5,38	3,16	6,05
**ADA**	84	0	6,50	5,93	6,22	6,32	5,89	6,06	5,79	7,07	5,37	6,22	5,51	6,78
**CCL20**	84	0	12,16	11,40	11,78	12,52	11,80	12,26	12,03	13,00	10,40	12,87	10,96	12,98
**NT3**	84	0	2,00	1,70	1,85	1,96	1,60	1,79	1,48	2,31	1,20	1,98	1,28	2,24
**IL20RA**	84	0	1,20	0,99	1,09	1,14	0,93	1,05	0,92	1,40	0,78	1,17	0,81	1,34
**SLAMF1**	84	0	3,02	2,72	2,87	2,99	2,57	2,75	2,42	3,69	2,09	3,24	2,20	3,39
**MMP1**	84	0	14,13	13,66	13,90	14,27	13,73	14,03	13,41	15,16	13,12	14,47	13,27	14,79
**SIRT2**	84	0	5,77	5,19	5,48	5,95	4,96	5,39	4,58	6,74	4,41	5,99	4,50	6,51
**IFNgamma**	84	0	8,18	10,14	9,16	7,94	10,27	8,79	5,61	10,12	8,18	12,11	6,95	11,59
**TNFB**	84	0	3,48	4,07	3,77	3,41	4,11	3,70	2,72	4,18	3,49	4,44	3,00	4,32
**Flt3L**	84	0	8,19	9,04	8,61	8,31	8,91	8,59	7,32	9,22	8,48	9,45	7,94	9,44
**SCF**	84	0	8,16	8,54	8,35	8,29	8,91	8,65	7,52	8,78	7,89	9,41	7,58	9,12
CD8A	84	0	9,06	9,37	9,22	9,39	9,30	9,34	8,38	9,92	8,61	10,18	8,51	10,08
GDNF	84	0	2,30	2,02	2,16	2,14	1,87	1,94	1,70	2,57	1,52	2,40	1,64	2,50
CDCP1	84	0	3,96	3,77	3,87	3,89	3,68	3,75	3,38	4,41	3,13	4,36	3,31	4,39
CD244	84	0	5,95	5,95	5,95	5,82	6,06	5,88	5,48	6,38	5,62	6,40	5,48	6,39
IL7	84	0	1,71	1,80	1,76	1,74	1,68	1,70	1,04	2,14	1,34	2,02	1,26	2,12
OPG	84	0	11,59	11,34	11,46	11,77	11,40	11,60	10,97	12,12	10,94	11,87	10,94	12,01
uPA	84	0	10,36	10,12	10,24	10,47	10,01	10,32	9,84	10,80	9,67	10,51	9,68	10,63
CXCL11	84	0	8,74	9,15	8,95	8,32	9,28	8,76	7,55	9,85	8,29	9,83	7,69	9,84
AXIN1	84	0	3,35	3,04	3,20	3,16	2,82	3,03	2,47	3,97	2,31	3,68	2,39	3,80
TRAIL	84	0	6,02	6,24	6,13	5,99	6,28	6,08	5,35	6,62	5,72	6,60	5,59	6,63
CXCL9	84	0	9,08	8,67	8,88	8,88	8,67	8,80	8,01	10,47	7,73	9,50	7,79	9,79
PSM	84	0	8,57	8,07	8,32	8,98	8,36	8,43	7,78	9,76	7,76	8,84	7,76	9,45
CCL4	84	0	9,09	8,86	8,97	8,81	8,47	8,79	7,87	10,84	7,39	10,92	7,55	10,93
CD6	84	0	5,36	5,53	5,44	5,26	5,56	5,43	4,81	5,86	5,12	6,06	5,03	6,03
IL18	84	0	10,91	11,06	10,99	10,77	11,27	10,98	10,15	11,67	9,90	11,99	9,98	11,80
TGFalpha	84	0	5,44	5,14	5,29	5,62	5,30	5,40	4,46	6,52	4,53	5,72	4,49	5,91
MCP4	84	0	13,15	12,83	12,99	12,93	12,70	12,78	12,25	13,68	12,25	13,33	12,24	13,58
CCL11	84	0	7,91	7,64	7,77	7,87	7,58	7,68	7,46	8,42	7,08	8,11	7,31	8,27
TNFSF14	84	0	6,48	6,61	6,54	6,32	6,57	6,52	5,90	7,14	6,19	7,11	6,10	7,13
IL10RA	84	0	1,33	1,16	1,24	1,12	1,05	1,09	0,88	1,47	0,71	1,34	0,78	1,40
LIFR	84	0	4,04	3,98	4,01	3,98	3,94	3,97	3,83	4,35	3,64	4,25	3,72	4,26
CCL19	84	0	10,84	10,55	10,70	11,21	10,59	11,03	10,26	11,54	9,90	11,30	9,98	11,48
IL10RB	84	0	7,58	7,46	7,52	7,63	7,45	7,57	7,31	7,87	7,10	7,79	7,18	7,86
IL18R1	84	0	9,24	9,03	9,14	9,09	8,96	9,01	8,69	9,76	8,57	9,56	8,62	9,70
CXCL5	84	0	9,21	8,97	9,09	9,47	8,85	9,04	7,67	10,91	7,58	10,21	7,63	10,54
TRANCE	84	0	2,54	2,90	2,72	2,52	2,56	2,55	1,63	3,20	2,28	3,76	2,11	3,43
IL12B	84	0	6,96	7,48	7,22	7,09	7,45	7,28	5,53	8,03	6,50	8,16	6,02	8,09
MMP10	84	0	10,03	9,59	9,81	9,97	9,62	9,75	9,38	11,13	9,07	10,06	9,18	10,51
TNF	84	0	6,67	6,00	6,33	6,03	5,42	5,68	5,06	8,12	4,75	6,96	4,91	7,28
CCL23	84	0	12,22	12,08	12,15	12,39	12,29	12,33	12,11	12,59	11,80	12,43	11,84	12,51
CD5	84	0	6,64	6,57	6,61	6,52	6,49	6,52	6,15	6,98	6,16	6,98	6,15	6,99
CCL3	84	0	9,24	8,55	8,89	9,03	8,39	8,81	8,16	10,39	7,23	9,51	7,48	9,89
CXCL10	84	0	11,30	11,36	11,33	11,40	11,47	11,43	10,21	12,66	10,64	12,09	10,44	12,39
FourEBP1	84	0	10,03	9,86	9,95	10,11	9,97	9,99	9,58	10,51	9,47	10,31	9,54	10,40
CCL28	84	0	2,24	2,01	2,12	2,11	1,91	1,99	1,84	2,51	1,59	2,33	1,70	2,42
DNER	84	0	7,63	7,71	7,67	7,65	7,71	7,66	7,34	7,97	7,42	8,06	7,39	8,03
ENRAGE	84	0	4,30	4,51	4,40	4,34	4,47	4,39	3,76	4,83	4,23	4,86	4,06	4,86
FGF19	84	0	9,56	9,11	9,33	9,52	8,89	9,18	8,36	10,87	8,07	10,07	8,20	10,37
NRTN	84	0	0,52	0,40	0,46	0,39	0,29	0,35	0,17	0,72	-0,04	0,53	0,10	0,68
MCP2	84	0	10,95	10,60	10,77	10,36	10,02	10,28	9,47	12,58	9,13	11,63	9,36	12,00
CASP8	84	0	6,44	6,64	6,54	6,39	6,52	6,48	5,50	7,47	5,72	7,98	5,51	7,68
CX3CL1	84	0	4,91	4,76	4,84	4,89	4,54	4,83	3,96	5,70	3,67	5,79	3,74	5,75
TWEAK	84	0	7,49	7,51	7,50	7,47	7,51	7,48	7,01	7,73	7,24	7,82	7,17	7,80
ST1A1	83	1	2,19	1,78	1,99	1,95	1,57	1,91	0,98	3,19	0,47	3,06	0,84	3,18
CSF1	84	0	10,09	10,09	10,09	10,06	10,09	10,08	9,90	10,27	9,95	10,16	9,94	10,22

Supplementary Table 3. Odds ratios (OR) with 95% confidence intervals (CI) and p-values between 42 bacteremia cases and 42 controls on 82 Olink® inflammation panel biomarker.

Biomarker	OR	95% CI		p-value
IL20RA	4.34	1.15	16.38	0.031
CD40	4.21	1.76	10.06	0.001
HGF	4.08	1.84	9.08	0.001
IL15RA	3.85	1.66	8.93	0.002
FGF5	3.40	1.36	8.54	0.009
ARTN	3.05	1.04	8.98	0.043
LAPTGFbeat1	2.78	1.19	6.50	0.018
PDL1	2.44	1.32	4.50	0.004
IL17C	2.38	1.38	4.11	0.002
CXCL1	2.35	1.38	3.98	0.002
MCP3	2.33	1.40	3.88	0.001
CCL28	2.30	0.95	5.59	0.065
CCL25	2.24	1.21	4.14	0.010
VEGFA	2.23	1.12	4.47	0.023
CXCL6	2.14	1.32	3.49	0.002
IL10RB	2.06	0.69	6.18	0.197
IL24	2.01	1.24	3.28	0.005
ADA	1.90	1.08	3.36	0.026
OPG	1.89	0.91	3.93	0.088
STAMBP	1.84	1.14	2.97	0.013
CST5	1.83	1.09	3.06	0.022
IL8	1.78	1.24	2.54	0.002
MMP10	1.75	1.02	3.00	0.043
GDNF	1.74	0.85	3.56	0.129
uPA	1.74	0.88	3.43	0.112
FGF23	1.74	1.27	2.38	0.001
CCL23	1.73	0.71	4.19	0.225
NT3	1.72	0.92	3.22	0.091
TNFRSF9	1.71	1.08	2.70	0.022
MCP1	1.67	1.07	2.61	0.024
SLAMF1	1.66	1.53	2.62	0.122
CCL11	1.63	0.89	3.00	0.114
FourEBP1	1.63	0.77	3.47	0.204
SIRT2	1.57	1.04	2.38	0.031
IL18R1	1.54	0.82	2.89	0.180
IL10RA	1.51	0.75	3.03	0.251
IL20	1.48	0.87	2.52	0.148
AXIN1	1.45	0.89	2.38	0.141
IL17A	1.43	1.07	1.91	0.017
IL6	1.41	1.32	3.49	0.002
CCL19	1.40	0.87	2.24	0.167
MCP4	1.38	0.88	2.18	0.162
NRTN	1.38	0.68	2.81	0.371

CCL20	1.37	1.01	1.87	0.046
LIF	1.35	1.07	1.69	0.011
CCL3	1.34	0.99	1.80	0.059
TGFalpha	1.33	0.86	2.05	0.204
MMP1	1.32	0.93	1.86	0.116
IL10	1.31	1.07	1.61	0.018
FGF21	1.30	1.06	1.60	0.012
FGF19	1.30	0.92	1.85	0.141
PSM	1.30	0.92	1.83	0.131
TNF	1.27	0.97	1.67	0.088
CDCP1	1.24	0.78	1.99	0.364
LIFR	1.24	0.54	2.83	0.608
ST1A1	1.23	0.91	1.65	0.182
CXCL9	1.20	0.89	1.63	0.225
CSF1	1.19	0.18	7.86	0.860
MCP2	1.18	0.87	1.60	0.283
CX3CL1	1.15	0.76	1.73	0.508
CD5	1.14	0.62	2.12	0.673
CCL4	1.09	0.84	1.42	0.525
CXCL5	1.08	0.85	1.37	0.553
CD244	0.99	0.51	1.93	0.978
CXCL10	0.97	0.69	1.35	0.838
TWEAK	0.94	0.46	1.91	0.859
CASP8	0.89	0.64	1.23	0.473
TNFSF14	0.87	0.55	1.37	0.545
IL7	0.87	0.50	1.52	0.614
CXCL11	0.86	0.66	1.12	0.268
IL18	0.86	0.55	1.33	0.488
ENRAGE	0.85	0.58	1.25	0.409
IFNgamma	0.84	0.72	0.97	0.014
CD8A	0.80	0.54	1.18	0.254
CD6	0.80	0.48	1.32	0.377
IL12B	0.78	0.57	1.07	0.126
TRAIL	0.72	0.41	1.25	0.241
TRANCE	0.68	0.42	1.10	0.113
SCF	0.67	0.42	1.07	0.091
DNER	0.62	0.21	1.80	0.378
Flt3L	0.59	0.39	0.89	0.012
TNFB	0.56	0.34	0.91	0.019

Supplementary Table 4. P-values and Benjamini-Hochberg P-values

Biomarker	P-values	Benjamini-Hochberg P-value
CD40	0,001	0,0164
HGF	0,001	0,0164
MCP3	0,001	0,0164
FGF23	0,001	0,0164
IL15RA	0,002	0,0164
IL17C	0,002	0,0164
CXCL1	0,002	0,0164
CXCL6	0,002	0,0164
IL8	0,002	0,0164
IL6	0,002	0,0164
PDL1	0,004	0,029818182
IL24	0,005	0,034166667
FGF5	0,009	0,056769231
CCL25	0,01	0,057882353
LIF	0,011	0,057882353
FGF21	0,012	0,057882353
Flt3L	0,012	0,057882353
STAMBP	0,013	0,059222222
IFNgamma	0,014	0,060421053
IL17A	0,017	0,067090909
LAPTGFbeat1	0,018	0,067090909
IL10	0,018	0,067090909
TNFB	0,019	0,06773913
CST5	0,022	0,07216
TNFRSF9	0,022	0,07216
VEGFA	0,023	0,072538462
MCP1	0,024	0,072888889
ADA	0,026	0,076142857
IL20RA	0,031	0,084733333
SIRT2	0,031	0,084733333
ARTN	0,043	0,1101875
MMP10	0,043	0,1101875
CCL20	0,046	0,11430303
CCL3	0,059	0,142294118
CCL28	0,065	0,152285714

OPG	0,088	0,191333333
TNF	0,088	0,191333333
NT3	0,091	0,191333333
SCF	0,091	0,191333333
uPA	0,112	0,221209302
TRANCE	0,113	0,221209302
CCL11	0,114	0,221209302
MMP1	0,116	0,221209302
SLAMF1	0,122	0,227363636
IL12B	0,126	0,228553191
GDNF	0,129	0,228553191
PSM	0,131	0,228553191
AXIN1	0,141	0,235959184
FGF19	0,141	0,235959184
IL20	0,148	0,24272
MCP4	0,162	0,260470588
CCL19	0,167	0,263346154
IL18R1	0,18	0,27637037
ST1A1	0,182	0,27637037
IL10RB	0,197	0,293473684
FourEBP1	0,204	0,293473684
TGFalpha	0,204	0,293473684
CCL23	0,225	0,312711864
CXCL9	0,225	0,312711864
TRAIL	0,241	0,329366667
IL10RA	0,251	0,335935484
CD8A	0,254	0,335935484
CXCL11	0,268	0,348825397
MCP2	0,283	0,36259375
CDCP1	0,364	0,455823529
NRTN	0,371	0,455823529
CD6	0,377	0,455823529
DNER	0,378	0,455823529
ENRAGE	0,409	0,486057971
CASP8	0,473	0,554085714
IL18	0,488	0,563605634
CX3CL1	0,508	0,578555556
CCL4	0,525	0,589726027

TNFSF14	0,545	0,603918919
CXCL5	0,553	0,604613333
LIFR	0,608	0,65387013
IL7	0,614	0,65387013
CD5	0,673	0,707512821
CXCL10	0,838	0,869822785
TWEAK	0,859	0,870617284
CSF1	0,86	0,870617284
CD244	0,978	0,978

Supplementary Table 5. Panther (v. 13.0) overrepresentation analysis was performed on the target proteins (30 analytes) and background proteins (49 analytes), and specific biological processes were identified using InteractiVenn (https://www.interactivenn.net/). Enriched processes are listed according to their FDR values. Note that exact enrichment values are not provided for the comparison of target and background proteins, as these processes are represented by two separate values from the analyses. FDR=False discovery rate, N/A=Data not available.

Overrepresented biological processes identified with the target set of proteins (FDR<0.05)

**Biological process**	**Number of proteins**	**Fold enrichment**	**Raw P value**	**FDR**
cellular response to oxygen-containing compound	13	8.83	4.88E-10	1.45E-07
negative regulation of cell population proliferation	11	10.78	1.97E-09	5.06E-07
cellular response to growth factor stimulus	9	13.08	1.52E-08	3.19E-06
response to growth factor	9	12.27	2.63E-08	4.90E-06
cellular response to fibroblast growth factor stimulus	5	40.83	1.32E-07	1.96E-05
positive regulation of B cell activation	5	40.35	1.40E-07	2.04E-05
response to fibroblast growth factor	5	37.28	2.09E-07	2.92E-05
positive regulation of epithelial cell apoptotic process	4	76.22	2.09E-07	2.90E-05
regulation of chemokine production	5	36.49	2.32E-07	3.11E-05
regulation of epithelial cell apoptotic process	5	33.96	3.33E-07	4.16E-05
positive regulation of programmed cell death	8	10.41	6.25E-07	7.15E-05
regulation of endothelial cell apoptotic process	4	53.8	8.74E-07	9.51E-05
induction of positive chemotaxis	3	> 100	1.01E-06	1.08E-04
positive regulation of immunoglobulin production	4	50.81	1.10E-06	1.15E-04
regulation of B cell activation	5	26.38	1.17E-06	1.20E-04
cell surface receptor signaling pathway via STAT	4	49.89	1.19E-06	1.21E-04
liver development	5	25.79	1.31E-06	1.32E-04
hepaticobiliary system development	5	25.22	1.46E-06	1.42E-04
negative regulation of response to stimulus	12	4.97	1.55E-06	1.50E-04
animal organ development	15	3.62	2.44E-06	2.20E-04
regulation of interleukin-6 production	5	22.57	2.53E-06	2.27E-04
positive regulation of endothelial cell apoptotic process	3	> 100	2.67E-06	2.39E-04
regulation of vasculature development	6	14	3.59E-06	3.09E-04
positive regulation of vasculature development	5	20.66	3.89E-06	3.29E-04
cellular response to interleukin-1	4	36.59	4.15E-06	3.49E-04
regulation of immunoglobulin production	4	36.11	4.38E-06	3.65E-04
epithelial cell proliferation	5	19.71	4.90E-06	4.02E-04
positive regulation of positive chemotaxis	3	82.32	6.29E-06	4.93E-04
regulation of positive chemotaxis	3	79.15	7.10E-06	5.43E-04
negative regulation of cell-cell adhesion	5	17.59	8.53E-06	6.38E-04
leukocyte homeostasis	4	28.58	1.11E-05	8.19E-04
positive regulation of cell division	4	28.29	1.16E-05	8.49E-04
liver regeneration	3	66.39	1.22E-05	8.84E-04
positive regulation of DNA-templated transcription	11	4.5	1.30E-05	9.39E-04
response to interleukin-1	4	27.44	1.31E-05	9.35E-04
positive regulation of RNA biosynthetic process	11	4.49	1.33E-05	9.46E-04
response to endogenous stimulus	10	4.99	1.50E-05	1.06E-03
regulation of gliogenesis	4	26.13	1.58E-05	1.12E-03
negative regulation of response to external stimulus	6	10.72	1.65E-05	1.16E-03
cellular response to endogenous stimulus	9	5.55	1.94E-05	1.34E-03

positive regulation of MAPKKK cascade by fibroblast growth factor receptor signaling pathway	2	> 100	2.05E-05	1.41E-03
positive regulation of morphogenesis of an epithelium	3	55.62	2.10E-05	1.44E-03
regulation of isotype switching	3	54.16	2.28E-05	1.55E-03
positive regulation of RNA metabolic process	11	4.18	2.61E-05	1.77E-03
negative regulation of apoptotic process	8	6.21	2.83E-05	1.90E-03
positive regulation of interleukin-10 production	3	50.2	2.87E-05	1.92E-03
gland development	6	9.71	2.88E-05	1.92E-03
regulation of primary miRNA processing	2	> 100	3.07E-05	2.03E-03
negative regulation of defense response	5	13.45	3.11E-05	2.04E-03
regulation of osteoblast differentiation	4	21.27	3.56E-05	2.30E-03
regulation of fat cell differentiation	4	20.63	4.01E-05	2.56E-03
embryonic organ development	6	9.11	4.12E-05	2.61E-03
positive regulation of transcription by RNA polymerase II	9	4.96	4.73E-05	2.87E-03
homeostasis of number of cells	5	12.25	4.85E-05	2.93E-03
negative regulation of leukocyte cell-cell adhesion	4	19.6	4.90E-05	2.95E-03
regulation of immune response	8	5.7	5.20E-05	3.11E-03
negative regulation of leukocyte migration	3	41.16	5.23E-05	3.11E-03
response to hypoxia	5	11.95	5.45E-05	3.21E-03
regulation of angiogenesis	5	11.91	5.54E-05	3.25E-03
cell surface receptor signaling pathway via JAK-STAT	3	40.35	5.55E-05	3.24E-03
negative regulation of cell migration	5	11.87	5.64E-05	3.25E-03
germinal center B cell differentiation	2	> 100	5.72E-05	3.28E-03
Peyer's patch development	2	> 100	5.72E-05	3.26E-03
mucosa-associated lymphoid tissue development	2	> 100	5.72E-05	3.25E-03
negative regulation of immune response to tumor cell	2	> 100	5.72E-05	3.24E-03
negative regulation of response to tumor cell	2	> 100	5.72E-05	3.23E-03
negative regulation of cytokine production	5	11.83	5.73E-05	3.22E-03
animal organ morphogenesis	8	5.62	5.76E-05	3.23E-03
negative regulation of cell adhesion	5	11.79	5.82E-05	3.25E-03
negative regulation of gene expression	8	5.57	6.11E-05	3.39E-03
B cell proliferation	3	38.83	6.23E-05	3.45E-03
regulation of morphogenesis of a branching structure	3	38.83	6.23E-05	3.44E-03
response to nutrient levels	6	8.45	6.24E-05	3.43E-03
lung alveolus development	3	38.11	6.59E-05	3.60E-03
response to decreased oxygen levels	5	11.4	6.83E-05	3.72E-03
defense response to bacterium	5	11.36	6.94E-05	3.76E-03
positive regulation of blood vessel endothelial cell migration	3	37.42	6.97E-05	3.76E-03
positive regulation of nucleobase-containing compound metabolic process	11	3.76	7.03E-05	3.78E-03
negative regulation of cell motility	5	11.28	7.16E-05	3.84E-03
lung vasculature development	2	> 100	7.35E-05	3.93E-03
positive regulation of protein kinase C signaling	2	> 100	7.35E-05	3.91E-03
fibroblast growth factor receptor signaling pathway	3	36.75	7.35E-05	3.89E-03
regulation of lymphocyte apoptotic process	3	34.88	8.60E-05	4.51E-03
positive regulation of angiogenesis	4	16.83	8.85E-05	4.61E-03
regulation of interleukin-10 production	3	34.3	9.04E-05	4.70E-03
regulation of interleukin-23 production	2	> 100	9.18E-05	4.75E-03
negative regulation of inflammatory response	4	16.63	9.28E-05	4.79E-03
negative regulation of locomotion	5	10.65	9.39E-05	4.83E-03
response to oxygen levels	5	10.49	1.01E-04	5.16E-03
regulation of immunoglobulin mediated immune response	3	32.67	1.05E-04	5.27E-03
regulation of B cell mediated immunity	3	32.67	1.05E-04	5.26E-03
endothelial cell apoptotic process	2	> 100	1.12E-04	5.61E-03
regulation of B cell proliferation	3	31.66	1.15E-04	5.73E-03

negative regulation of cell communication	9	4.41	1.18E-04	5.85E-03
negative regulation of signaling	9	4.41	1.18E-04	5.86E-03
animal organ regeneration	3	30.72	1.26E-04	6.19E-03
negative regulation of muscle tissue development	2	> 100	1.34E-04	6.57E-03
positive regulation of osteoblast differentiation	3	29.83	1.37E-04	6.69E-03
positive regulation of gliogenesis	3	29.4	1.43E-04	6.96E-03
regulation of cell division	4	14.67	1.50E-04	7.26E-03
B cell activation	4	14.67	1.50E-04	7.24E-03
lung development	4	14.44	1.60E-04	7.67E-03
negative regulation of T cell proliferation	3	27.81	1.69E-04	8.06E-03
respiratory tube development	4	14.14	1.73E-04	8.23E-03
regulation of miRNA transcription	3	27.44	1.76E-04	8.33E-03
regulation of neurogenesis	5	9.25	1.82E-04	8.61E-03
regulation of glial cell differentiation	3	27.08	1.83E-04	8.61E-03
response to activity	3	27.08	1.83E-04	8.59E-03
inflammatory response to wounding	2	98	1.85E-04	8.66E-03
cellular response to hepatocyte growth factor stimulus	2	91.47	2.13E-04	9.95E-03
regulation of extracellular matrix disassembly	2	91.47	2.13E-04	9.92E-03
regulation of reproductive process	4	13.39	2.14E-04	9.92E-03
multicellular organismal-level homeostasis	6	6.7	2.22E-04	1.02E-02
response to vitamin	3	25.1	2.29E-04	1.05E-02
regulation of miRNA processing	2	85.75	2.43E-04	1.11E-02
regulation of miRNA metabolic process	3	24.21	2.55E-04	1.15E-02
regulation of lymphocyte differentiation	4	12.7	2.61E-04	1.18E-02
respiratory system development	4	12.65	2.66E-04	1.20E-02
regulation of regulatory ncRNA processing	2	80.71	2.76E-04	1.24E-02
response to hepatocyte growth factor	2	80.71	2.76E-04	1.23E-02
positive regulation of vascular permeability	2	80.71	2.76E-04	1.23E-02
regulation of leukocyte apoptotic process	3	23.39	2.82E-04	1.25E-02
negative regulation of lymphocyte proliferation	3	23.12	2.92E-04	1.29E-02
negative regulation of autophagy	3	22.87	3.01E-04	1.32E-02
regulation of exit from mitosis	2	76.22	3.10E-04	1.36E-02
positive regulation of branching involved in ureteric bud morphogenesis	2	76.22	3.10E-04	1.35E-02
regulation of protein kinase C signaling	2	76.22	3.10E-04	1.35E-02
positive regulation of lymphocyte apoptotic process	2	76.22	3.10E-04	1.35E-02
negative regulation of mononuclear cell proliferation	3	22.62	3.11E-04	1.35E-02
regulation of blood vessel endothelial cell migration	3	22.37	3.22E-04	1.38E-02
regulation of striated muscle tissue development	2	72.21	3.46E-04	1.47E-02
regulation of B cell apoptotic process	2	72.21	3.46E-04	1.47E-02
response to wounding	5	8	3.56E-04	1.51E-02
positive regulation of interleukin-6 production	3	21	3.87E-04	1.63E-02
negative regulation of leukocyte proliferation	3	21	3.87E-04	1.63E-02
regulation of phagocytosis	3	21	3.87E-04	1.62E-02
negative regulation of cellular component organization	6	6.03	3.93E-04	1.64E-02
positive regulation of neurogenesis	4	11.39	3.96E-04	1.65E-02
hematopoietic or lymphoid organ development	3	20.79	3.99E-04	1.66E-02
regulation of branching involved in ureteric bud morphogenesis	2	65.33	4.24E-04	1.75E-02
surfactant homeostasis	2	65.33	4.24E-04	1.75E-02
regulation of neural precursor cell proliferation	3	20.18	4.35E-04	1.78E-02
regulation of nervous system development	5	7.62	4.44E-04	1.81E-02
regulation of epithelial to mesenchymal transition	3	19.98	4.48E-04	1.82E-02
negative regulation of chemokine production	2	62.36	4.66E-04	1.88E-02
positive regulation of endothelial cell migration	3	19.6	4.74E-04	1.91E-02
positive regulation of tumor necrosis factor production	3	19.42	4.87E-04	1.95E-02

neurogenesis	8	4.11	4.99E-04	1.99E-02
response to salt	2	59.65	5.10E-04	2.03E-02
positive regulation of sprouting angiogenesis	2	59.65	5.10E-04	2.03E-02
negative regulation of cell development	4	10.47	5.42E-04	2.15E-02
positive regulation of tumor necrosis factor superfamily cytokine production	3	18.71	5.43E-04	2.15E-02
regulation of macrophage differentiation	2	57.17	5.56E-04	2.19E-02
positive regulation of signaling receptor activity	2	57.17	5.56E-04	2.18E-02
regulation of muscle organ development	2	57.17	5.56E-04	2.18E-02
regulation of immune response to tumor cell	2	57.17	5.56E-04	2.17E-02
adaptive immune response based on somatic recombination of immune receptors built from immunoglobulin superfamily domains	4	10.32	5.74E-04	2.24E-02
negative regulation of cellular catabolic process	3	18.05	6.03E-04	2.34E-02
negative regulation of neural precursor cell proliferation	2	54.88	6.04E-04	2.33E-02
regulation of response to tumor cell	2	54.88	6.04E-04	2.33E-02
defense response to protozoan	2	52.77	6.53E-04	2.50E-02
regulation of peptidyl-serine phosphorylation	3	17.15	7.00E-04	2.66E-02
system development	13	2.53	7.05E-04	2.67E-02
mature B cell differentiation involved in immune response	2	50.81	7.05E-04	2.66E-02
epithelial cell maturation	2	50.81	7.05E-04	2.66E-02
cellular response to interleukin-6	2	50.81	7.05E-04	2.65E-02
negative regulation of miRNA transcription	2	50.81	7.05E-04	2.64E-02
regulation of DNA metabolic process	5	6.79	7.48E-04	2.80E-02
negative regulation of immune effector process	3	16.73	7.52E-04	2.81E-02
regulation of endocytosis	4	9.59	7.53E-04	2.80E-02
negative regulation of miRNA metabolic process	2	49	7.58E-04	2.82E-02
positive regulation of interleukin-17 production	2	49	7.58E-04	2.81E-02
positive regulation of leukocyte apoptotic process	2	49	7.58E-04	2.80E-02
negative regulation of biomineral tissue development	2	49	7.58E-04	2.80E-02
regulation of miRNA-mediated gene silencing	2	49	7.58E-04	2.79E-02
positive regulation of nervous system development	4	9.43	8.03E-04	2.93E-02
response to vitamin D	2	47.31	8.14E-04	2.97E-02
response to protozoan	2	47.31	8.14E-04	2.96E-02
regulation of astrocyte differentiation	2	47.31	8.14E-04	2.95E-02
regulation of post-transcriptional gene silencing by regulatory ncRNA	2	47.31	8.14E-04	2.94E-02
neutrophil activation	2	47.31	8.14E-04	2.94E-02
positive regulation of isotype switching	2	47.31	8.14E-04	2.93E-02
cellular response to hypoxia	3	16.2	8.25E-04	2.96E-02
positive regulation of DNA metabolic process	4	9.33	8.34E-04	2.99E-02
response to abiotic stimulus	7	4.37	8.42E-04	3.01E-02
negative regulation of T cell activation	3	15.95	8.63E-04	3.08E-02
B cell differentiation	3	15.95	8.63E-04	3.07E-02
positive regulation of extracellular matrix organization	2	45.73	8.71E-04	3.08E-02
regulation of post-transcriptional gene silencing	2	45.73	8.71E-04	3.08E-02
cellular response to low-density lipoprotein particle stimulus	2	45.73	8.71E-04	3.07E-02
response to lipoprotein particle	2	45.73	8.71E-04	3.06E-02
positive regulation of vascular endothelial growth factor production	2	45.73	8.71E-04	3.06E-02
negative regulation of protein metabolic process	6	5.16	8.86E-04	3.09E-02
negative regulation of neurogenesis	3	15.71	9.02E-04	3.13E-02
regulation of response to biotic stimulus	5	6.48	9.22E-04	3.18E-02
digestive tract development	3	15.59	9.23E-04	3.18E-02

regulation of leukocyte adhesion to vascular endothelial cell	2	44.26	9.30E-04	3.20E-02
regulation of gene silencing by regulatory ncRNA	2	44.26	9.30E-04	3.19E-02
cellular response to decreased oxygen levels	3	15.36	9.64E-04	3.29E-02
regulation of collagen biosynthetic process	2	42.88	9.92E-04	3.38E-02
response to interleukin-6	2	42.88	9.92E-04	3.37E-02
response to glucocorticoid	3	15.13	1.01E-03	3.39E-02
negative regulation of nervous system development	3	15.02	1.03E-03	3.45E-02
mature B cell differentiation	2	41.58	1.05E-03	3.53E-02
cellular response to lipoprotein particle stimulus	2	41.58	1.05E-03	3.52E-02
embryonic digestive tract development	2	41.58	1.05E-03	3.51E-02
regulation of vascular endothelial growth factor production	2	41.58	1.05E-03	3.50E-02
regulation of cell shape	3	14.81	1.07E-03	3.55E-02
vasculature development	5	6.26	1.08E-03	3.57E-02
granulocyte activation	2	40.35	1.12E-03	3.70E-02
negative regulation of cytokine production involved in immune response	2	40.35	1.12E-03	3.69E-02
wound healing	4	8.55	1.16E-03	3.80E-02
response to organic cyclic compound	6	4.91	1.16E-03	3.79E-02
regulation of DNA recombination	3	14.39	1.16E-03	3.80E-02
digestive system development	3	14.39	1.16E-03	3.80E-02
regulation of meiotic nuclear division	2	39.2	1.19E-03	3.87E-02
regulation of kidney development	2	39.2	1.19E-03	3.86E-02
intracellular chemical homeostasis	5	6.03	1.28E-03	4.14E-02
placenta development	3	13.81	1.31E-03	4.24E-02
cellular response to oxygen levels	3	13.81	1.31E-03	4.23E-02
regulation of collagen metabolic process	2	37.08	1.33E-03	4.27E-02
cellular response to tumor necrosis factor	3	13.72	1.33E-03	4.29E-02
positive regulation of epithelial cell migration	3	13.72	1.33E-03	4.28E-02
positive regulation of endocytosis	3	13.45	1.41E-03	4.49E-02
negative regulation of penile erection	1	> 100	1.46E-03	4.62E-02
xanthine biosynthetic process	1	> 100	1.46E-03	4.61E-02
negative regulation of adenosine receptor signaling pathway	1	> 100	1.46E-03	4.60E-02
positive regulation of cell migration by vascular endothelial growth factor signaling pathway	1	> 100	1.46E-03	4.59E-02
positive regulation of interleukin-16 production	1	> 100	1.46E-03	4.58E-02
positive regulation of endothelial cell chemotaxis by VEGF-activated vascular endothelial growth factor receptor signaling pathway	1	> 100	1.46E-03	4.57E-02
positive regulation of microglia differentiation	1	> 100	1.46E-03	4.56E-02
regulation of microglia differentiation	1	> 100	1.46E-03	4.55E-02
regulation of interleukin-16 production	1	> 100	1.46E-03	4.54E-02
negative regulation of oligodendrocyte progenitor proliferation	1	> 100	1.46E-03	4.54E-02
positive regulation of activated CD8-positive, alpha-beta T cell apoptotic process	1	> 100	1.46E-03	4.53E-02
glucagon secretion	1	> 100	1.46E-03	4.52E-02
cellular response to caloric restriction	1	> 100	1.46E-03	4.51E-02
mature B cell apoptotic process	1	> 100	1.46E-03	4.50E-02
negative regulation of chronic inflammatory response to antigenic stimulus	1	> 100	1.46E-03	4.49E-02
maintenance of blood-brain barrier	2	35.18	1.47E-03	4.51E-02
negative regulation of ossification	2	35.18	1.47E-03	4.51E-02
response to corticosteroid	3	13.03	1.55E-03	4.72E-02
response to nutrient	3	13.03	1.55E-03	4.71E-02
calcium-mediated signaling	3	12.86	1.60E-03	4.86E-02

regulation of secretion	5	5.72	1.61E-03	4.88E-02
regulation of interleukin-17 production	2	33.46	1.63E-03	4.91E-02
regulation of p38MAPK cascade	2	33.46	1.63E-03	4.90E-02
regulation of T cell apoptotic process	2	33.46	1.63E-03	4.89E-02
regulation of sprouting angiogenesis	2	33.46	1.63E-03	4.88E-02
negative regulation of neuron apoptotic process	3	12.78	1.63E-03	4.89E-02
regeneration	3	12.78	1.63E-03	4.88E-02
regulation of body fluid levels	4	7.75	1.66E-03	4.93E-02
response to organonitrogen compound	6	4.56	1.68E-03	4.97E-02

Overrepresented biological processes identified with the background set of proteins (FDR<0.05)

Biological process	Number of proteins	Fold enrichment	Raw P value	FDR
regulation of osteoclast differentiation	7	40.02	4.21E-10	6.71E-08
regulation of myeloid cell differentiation	9	18.71	1.05E-09	1.57E-07
lymphocyte chemotaxis	5	82.32	3.53E-09	4.52E-07
natural killer cell activation	6	40.49	7.56E-09	8.94E-07
positive regulation of osteoclast differentiation	5	70.97	7.83E-09	9.18E-07
extrinsic apoptotic signaling pathway	7	25.73	9.63E-09	1.10E-06
regulation of T-helper 1 type immune response	5	66.39	1.12E-08	1.25E-06
positive regulation of non-canonical NF-kappaB signal transduction	6	37.99	1.12E-08	1.24E-06
T cell chemotaxis	4	> 100	2.17E-08	2.34E-06
positive regulation of type II interferon production	6	32.07	3.14E-08	3.29E-06
positive regulation of inflammatory response	7	19.34	6.97E-08	6.75E-06
positive regulation of T-helper 1 type immune response	4	91.47	9.20E-08	8.75E-06
regulation of non-canonical NF-kappaB signal transduction	6	26.27	1.04E-07	9.74E-06
positive regulation of JNK cascade	6	25.46	1.26E-07	1.15E-05
positive regulation of T cell proliferation	6	24.21	1.70E-07	1.55E-05
response to virus	9	9.9	2.55E-07	2.28E-05
positive regulation of ERK1 and ERK2 cascade	7	15.49	3.16E-07	2.77E-05
positive regulation of calcium ion transport	6	21.29	3.65E-07	3.16E-05
regulation of type II interferon production	6	20.93	4.04E-07	3.47E-05
positive regulation of macrophage migration	4	63.32	4.43E-07	3.76E-05
positive regulation of microglial cell migration	3	> 100	4.69E-07	3.96E-05
positive regulation of transport	12	5.92	5.39E-07	4.52E-05
regulation of sequestering of calcium ion	6	18.85	7.49E-07	6.12E-05
positive regulation of T-helper 1 cell cytokine production	3	> 100	7.49E-07	6.09E-05
regulation of T-helper 1 cell cytokine production	3	> 100	7.49E-07	6.06E-05
regulation of microglial cell migration	3	> 100	7.49E-07	6.03E-05
positive regulation of canonical NF-kappaB signal transduction	7	13.53	7.89E-07	6.31E-05
apoptotic signaling pathway	8	10.42	8.67E-07	6.90E-05
regulation of myoblast differentiation	5	28.19	9.14E-07	7.24E-05
T cell activation	8	10.26	9.75E-07	7.64E-05
regulation of JNK cascade	6	17.77	1.06E-06	8.22E-05
multicellular organismal process	32	2.11	1.20E-06	9.29E-05
innate immune response	11	6.07	1.35E-06	1.03E-04
negative regulation of extrinsic apoptotic signaling pathway in absence of ligand	4	48.42	1.35E-06	1.02E-04
negative regulation of signal transduction in absence of ligand	4	48.42	1.35E-06	1.02E-04

regulation of extrinsic apoptotic signaling pathway	6	16.35	1.72E-06	1.28E-04
positive regulation of protein kinase activity	7	11.81	1.95E-06	1.44E-04
positive regulation of glial cell migration	3	> 100	2.20E-06	1.60E-04
regulation of interleukin-1 beta production	5	21.89	3.22E-06	2.30E-04
positive regulation of lymphocyte migration	4	39.2	3.22E-06	2.30E-04
positive regulation of release of sequestered calcium ion into cytosol	4	39.2	3.22E-06	2.29E-04
regulation of macrophage migration	4	38.29	3.55E-06	2.51E-04
regulation of extrinsic apoptotic signaling pathway in absence of ligand	4	37.42	3.90E-06	2.73E-04
positive regulation of mitotic nuclear division	4	37.42	3.90E-06	2.71E-04
positive regulation of phosphatidylinositol 3-kinase/protein kinase B signal transduction	6	13.64	4.91E-06	3.36E-04
regulation of canonical NF-kappaB signal transduction	7	10.18	5.20E-06	3.51E-04
positive regulation of neuroinflammatory response	3	82.32	6.01E-06	4.04E-04
positive regulation of kinase activity	7	9.94	6.10E-06	4.08E-04
G protein-coupled receptor signaling pathway	13	4.26	6.45E-06	4.30E-04
positive chemotaxis	4	32.93	6.54E-06	4.34E-04
myeloid cell differentiation	7	9.73	6.98E-06	4.59E-04
intracellular calcium ion homeostasis	6	12.41	8.45E-06	5.44E-04
leukocyte activation involved in immune response	6	12.41	8.45E-06	5.42E-04
cell activation involved in immune response	6	12.17	9.47E-06	6.04E-04
positive regulation of monoatomic ion transport	6	12.05	1.00E-05	6.36E-04
cell development	17	3.14	1.06E-05	6.70E-04
osteoclast differentiation	4	28.39	1.19E-05	7.48E-04
leukocyte migration involved in inflammatory response	3	64.99	1.27E-05	7.95E-04
positive regulation of lymphocyte chemotaxis	3	64.99	1.27E-05	7.92E-04
regulation of glial cell migration	3	64.99	1.27E-05	7.88E-04
positive regulation of interleukin-1 beta production	4	27.44	1.36E-05	8.39E-04
positive regulation of nuclear division	4	26.99	1.45E-05	8.82E-04
regulation of myoblast fusion	3	61.74	1.49E-05	9.04E-04
mammary duct terminal end bud growth	2	> 100	1.73E-05	1.02E-03
positive regulation of NF-kappaB transcription factor activity	5	15.36	1.82E-05	1.07E-03
immune effector process	8	6.87	1.85E-05	1.07E-03
T-helper 1 type immune response	3	53.69	2.31E-05	1.33E-03
positive regulation of T cell mediated immunity	4	23.52	2.51E-05	1.44E-03
positive regulation of transferase activity	7	7.98	2.52E-05	1.44E-03
regulation of calcium ion transport	6	10.16	2.62E-05	1.49E-03
positive regulation of interleukin-1 production	4	22.87	2.81E-05	1.59E-03
negative regulation of myoblast differentiation	3	49.39	2.99E-05	1.67E-03
positive regulation of T-helper cell differentiation	3	49.39	2.99E-05	1.67E-03
positive regulation of activated T cell proliferation	3	49.39	2.99E-05	1.66E-03
positive regulation of T cell cytokine production	3	47.49	3.37E-05	1.84E-03
tube morphogenesis	9	5.43	3.39E-05	1.85E-03
regulation of phosphatidylinositol 3-kinase/protein kinase B signal transduction	6	9.68	3.43E-05	1.86E-03
positive regulation of MAP kinase activity	4	21.11	3.85E-05	2.08E-03
regulation of syncytium formation by plasma membrane fusion	3	44.1	4.24E-05	2.26E-03
positive regulation of calcium ion transmembrane transport	4	20.33	4.47E-05	2.37E-03
regulation of release of sequestered calcium ion into cytosol	4	20.08	4.69E-05	2.46E-03
positive regulation of natural killer cell chemotaxis	2	> 100	5.76E-05	2.96E-03
osteoclast proliferation	2	> 100	5.76E-05	2.95E-03
inorganic ion homeostasis	7	6.67	7.84E-05	3.95E-03

negative regulation of extrinsic apoptotic signaling pathway	4	17.33	8.34E-05	4.19E-03
regulation of neuroinflammatory response	3	35.28	8.36E-05	4.19E-03
cellular response to interleukin-18	2	> 100	8.62E-05	4.30E-03
positive regulation of NK T cell proliferation	2	> 100	8.62E-05	4.29E-03
interleukin-18-mediated signaling pathway	2	> 100	8.62E-05	4.28E-03
positive regulation of I-kappaB phosphorylation	2	> 100	8.62E-05	4.26E-03
regulation of protein kinase activity	7	6.49	9.30E-05	4.58E-03
positive regulation of CD4-positive, alpha-beta T cell differentiation	3	33.37	9.89E-05	4.82E-03
regulation of T cell cytokine production	3	32.49	1.07E-04	5.21E-03
regulation of T cell mediated immunity	4	15.98	1.14E-04	5.53E-03
ameboidal-type cell migration	5	10.34	1.20E-04	5.76E-03
positive regulation of fever generation	2	> 100	1.21E-04	5.77E-03
regulation of NK T cell proliferation	2	> 100	1.21E-04	5.75E-03
mast cell migration	2	> 100	1.21E-04	5.73E-03
regulation of I-kappaB phosphorylation	2	> 100	1.21E-04	5.71E-03
gland morphogenesis	4	15.68	1.23E-04	5.81E-03
regulation of activated T cell proliferation	3	30.12	1.35E-04	6.33E-03
positive regulation of transmembrane transport	5	9.94	1.44E-04	6.73E-03
negative regulation of cell differentiation	8	5.09	1.53E-04	7.11E-03
T cell costimulation	3	28.72	1.56E-04	7.21E-03
extrinsic apoptotic signaling pathway via death domain receptors	3	28.72	1.56E-04	7.19E-03
positive regulation of receptor signaling pathway via JAK-STAT	3	28.72	1.56E-04	7.17E-03
response to interleukin-18	2	> 100	1.60E-04	7.37E-03
regulation of T-helper cell differentiation	3	28.06	1.67E-04	7.63E-03
lymphocyte costimulation	3	28.06	1.67E-04	7.61E-03
positive regulation of DNA-binding transcription factor activity	5	9.57	1.72E-04	7.82E-03
MAPK cascade	5	9.44	1.83E-04	8.29E-03
positive regulation of CD4-positive, alpha-beta T cell activation	3	26.84	1.90E-04	8.56E-03
regulation of mitotic nuclear division	4	13.84	1.99E-04	8.94E-03
monoatomic cation homeostasis	7	5.72	2.03E-04	9.10E-03
regulation of fever generation	2	91.47	2.06E-04	9.13E-03
positive regulation of NK T cell activation	2	91.47	2.06E-04	9.11E-03
microglial cell proliferation	2	91.47	2.06E-04	9.08E-03
macrophage proliferation	2	91.47	2.06E-04	9.05E-03
regulation of kinase activity	7	5.69	2.08E-04	9.13E-03
positive regulation of T cell differentiation	4	13.61	2.12E-04	9.26E-03
positive regulation of organelle organization	7	5.66	2.16E-04	9.39E-03
negative regulation of interleukin-6 production	3	25.73	2.16E-04	9.36E-03
positive regulation of protein serine/threonine kinase activity	4	13.39	2.26E-04	9.75E-03
monoatomic ion homeostasis	7	5.61	2.29E-04	9.85E-03
positive regulation of extrinsic apoptotic signaling pathway	3	24.7	2.44E-04	1.04E-02
mammary gland development	4	13.07	2.48E-04	1.06E-02
regulation of MAP kinase activity	4	12.96	2.56E-04	1.09E-02
positive regulation of natural killer cell proliferation	2	82.32	2.57E-04	1.09E-02
positive regulation of heat generation	2	82.32	2.57E-04	1.09E-02
regulation of granulocyte chemotaxis	3	24.21	2.59E-04	1.09E-02
regulation of neuron apoptotic process	5	8.61	2.80E-04	1.18E-02
regulation of apoptotic signaling pathway	6	6.59	2.84E-04	1.19E-02
regulation of metal ion transport	6	6.53	2.96E-04	1.23E-02

positive regulation of tissue remodeling	2	74.84	3.14E-04	1.30E-02
regulation of NK T cell activation	2	74.84	3.14E-04	1.30E-02
positive regulation of cation transmembrane transport	4	12.2	3.23E-04	1.33E-02
neural crest cell migration	3	22.45	3.24E-04	1.33E-02
positive regulation of alpha-beta T cell differentiation	3	22.45	3.24E-04	1.32E-02
positive regulation of cell-matrix adhesion	3	22.05	3.42E-04	1.38E-02
mesenchymal cell migration	3	21.66	3.60E-04	1.45E-02
positive regulation of calcium-mediated signaling	3	21.66	3.60E-04	1.45E-02
regulation of CD4-positive, alpha-beta T cell differentiation	3	21.66	3.60E-04	1.44E-02
chronic inflammatory response	2	68.6	3.76E-04	1.50E-02
regulation of natural killer cell proliferation	2	68.6	3.76E-04	1.50E-02
interleukin-10-mediated signaling pathway	2	68.6	3.76E-04	1.50E-02
response to macrophage colony-stimulating factor	2	68.6	3.76E-04	1.49E-02
regulation of odontogenesis of dentin-containing tooth	2	68.6	3.76E-04	1.49E-02
mammary gland epithelium development	3	20.93	3.99E-04	1.57E-02
regulation of cytokine production involved in inflammatory response	3	20.58	4.19E-04	1.65E-02
immunological synapse formation	2	63.32	4.44E-04	1.74E-02
regulation of heat generation	2	63.32	4.44E-04	1.74E-02
glial cell-derived neurotrophic factor receptor signaling pathway	2	63.32	4.44E-04	1.73E-02
regulation of nuclear division	4	11.2	4.46E-04	1.74E-02
positive regulation of monoatomic ion transmembrane transport	4	11.12	4.57E-04	1.78E-02
defense response to virus	5	7.54	5.15E-04	1.99E-02
negative regulation by host of viral transcription	2	58.8	5.17E-04	1.99E-02
myeloid cell activation involved in immune response	3	19	5.30E-04	2.04E-02
positive regulation of neuron apoptotic process	3	19	5.30E-04	2.03E-02
response to radiation	6	5.82	5.45E-04	2.08E-02
intracellular monoatomic cation homeostasis	6	5.77	5.73E-04	2.17E-02
positive regulation of granulocyte macrophage colony-stimulating factor production	2	54.88	5.95E-04	2.26E-02
positive regulation of myoblast fusion	2	54.88	5.95E-04	2.25E-02
regulation of T cell chemotaxis	2	54.88	5.95E-04	2.24E-02
blood vessel morphogenesis	6	5.72	6.01E-04	2.26E-02
regulation of transferase activity	7	4.73	6.35E-04	2.38E-02
intracellular monoatomic ion homeostasis	6	5.65	6.38E-04	2.38E-02
regulation of cytokine-mediated signaling pathway	4	10.1	6.58E-04	2.45E-02
regulation of calcium ion transmembrane transport	4	9.86	7.20E-04	2.66E-02
regulation of monoatomic ion transport	6	5.5	7.36E-04	2.71E-02
positive regulation of alpha-beta T cell activation	3	16.92	7.45E-04	2.73E-02
regulation of receptor signaling pathway via JAK-STAT	3	16.92	7.45E-04	2.73E-02
T cell proliferation	3	16.69	7.75E-04	2.82E-02
regulation of alpha-beta T cell differentiation	3	16.46	8.06E-04	2.93E-02
regulation of transmembrane transport	6	5.38	8.25E-04	2.99E-02
regulation of response to cytokine stimulus	4	9.46	8.39E-04	3.04E-02
response to tumor necrosis factor	4	9.41	8.58E-04	3.09E-02
regulation of granulocyte macrophage colony-stimulating factor production	2	45.73	8.63E-04	3.11E-02
positive regulation of macrophage derived foam cell differentiation	2	45.73	8.63E-04	3.10E-02
positive regulation of Ras protein signal transduction	2	45.73	8.63E-04	3.09E-02
neural crest cell development	3	15.83	9.03E-04	3.23E-02
regulation of CD4-positive, alpha-beta T cell activation	3	15.63	9.37E-04	3.34E-02
cellular response to virus	3	15.63	9.37E-04	3.33E-02

regulation of odontogenesis	2	43.33	9.63E-04	3.42E-02
positive regulation of macrophage chemotaxis	2	43.33	9.63E-04	3.41E-02
T cell differentiation	4	9.05	9.92E-04	3.51E-02
positive regulation of cytokine production involved in immune response	3	15.24	1.01E-03	3.54E-02
cellular response to abiotic stimulus	5	6.45	1.04E-03	3.63E-02
cellular response to environmental stimulus	5	6.45	1.04E-03	3.62E-02
regulation of T cell differentiation	4	8.9	1.05E-03	3.66E-02
mammary gland lobule development	2	41.16	1.07E-03	3.71E-02
mammary gland alveolus development	2	41.16	1.07E-03	3.70E-02
cellular response to type II interferon	3	14.88	1.08E-03	3.73E-02
positive regulation of cell cycle	5	6.37	1.10E-03	3.78E-02
metabolic process	8	0.42	1.11E-03	3.81E-02
stem cell development	3	14.7	1.12E-03	3.84E-02
modulation by host of symbiont process	3	14.53	1.16E-03	3.95E-02
positive regulation of natural killer cell activation	2	39.2	1.18E-03	4.01E-02
branching involved in mammary gland duct morphogenesis	2	37.42	1.30E-03	4.36E-02
negative regulation of developmental process	8	3.68	1.32E-03	4.45E-02
regulation of gene expression	22	1.86	1.38E-03	4.63E-02
neural crest cell differentiation	3	13.57	1.41E-03	4.70E-02
positive regulation of syncytium formation by plasma membrane fusion	2	35.79	1.42E-03	4.71E-02
positive regulation of epithelial cell proliferation	4	8.19	1.43E-03	4.74E-02
regulation of DNA-binding transcription factor activity	5	5.95	1.49E-03	4.91E-02

Overrepresented biological processes identified with both the target set and the background set of proteins (FDR<0.05)

Biological process				
cell surface receptor signaling pathway	N/A	N/A	N/A	N/A
leukocyte migration	N/A	N/A	N/A	N/A
response to cytokine	N/A	N/A	N/A	N/A
response to peptide	N/A	N/A	N/A	N/A
cell chemotaxis	N/A	N/A	N/A	N/A
regulation of locomotion	N/A	N/A	N/A	N/A
cellular response to cytokine stimulus	N/A	N/A	N/A	N/A
inflammatory response	N/A	N/A	N/A	N/A
signal transduction	N/A	N/A	N/A	N/A
regulation of cell migration	N/A	N/A	N/A	N/A
regulation of cell motility	N/A	N/A	N/A	N/A
signaling	N/A	N/A	N/A	N/A
cytokine-mediated signaling pathway	N/A	N/A	N/A	N/A
cell communication	N/A	N/A	N/A	N/A
regulation of immune system process	N/A	N/A	N/A	N/A
cell migration	N/A	N/A	N/A	N/A
cellular response to chemical stimulus	N/A	N/A	N/A	N/A
regulation of cell population proliferation	N/A	N/A	N/A	N/A
chemotaxis	N/A	N/A	N/A	N/A
taxis	N/A	N/A	N/A	N/A
positive regulation of immune system process	N/A	N/A	N/A	N/A
locomotion	N/A	N/A	N/A	N/A
cell motility	N/A	N/A	N/A	N/A
cellular response to lipopolysaccharide	N/A	N/A	N/A	N/A
cellular response to molecule of bacterial origin	N/A	N/A	N/A	N/A

immune system process	N/A	N/A	N/A	N/A
positive regulation of locomotion	N/A	N/A	N/A	N/A
myeloid leukocyte migration	N/A	N/A	N/A	N/A
cellular response to stimulus	N/A	N/A	N/A	N/A
regulation of leukocyte migration	N/A	N/A	N/A	N/A
leukocyte chemotaxis	N/A	N/A	N/A	N/A
chemokine-mediated signaling pathway	N/A	N/A	N/A	N/A
cellular response to biotic stimulus	N/A	N/A	N/A	N/A
response to chemical	N/A	N/A	N/A	N/A
cellular response to chemokine	N/A	N/A	N/A	N/A
response to chemokine	N/A	N/A	N/A	N/A
regulation of response to external stimulus	N/A	N/A	N/A	N/A
granulocyte migration	N/A	N/A	N/A	N/A
cellular response to lipid	N/A	N/A	N/A	N/A
antimicrobial humoral immune response mediated by antimicrobial peptide	N/A	N/A	N/A	N/A
response to external stimulus	N/A	N/A	N/A	N/A
positive regulation of response to stimulus	N/A	N/A	N/A	N/A
response to other organism	N/A	N/A	N/A	N/A
response to external biotic stimulus	N/A	N/A	N/A	N/A
positive regulation of cell migration	N/A	N/A	N/A	N/A
immune response	N/A	N/A	N/A	N/A
response to biotic stimulus	N/A	N/A	N/A	N/A
positive regulation of cell population proliferation	N/A	N/A	N/A	N/A
positive regulation of cell motility	N/A	N/A	N/A	N/A
response to lipopolysaccharide	N/A	N/A	N/A	N/A
defense response to other organism	N/A	N/A	N/A	N/A
biological process involved in interspecies interaction between organisms	N/A	N/A	N/A	N/A
response to molecule of bacterial origin	N/A	N/A	N/A	N/A
regulation of multicellular organismal process	N/A	N/A	N/A	N/A
antimicrobial humoral response	N/A	N/A	N/A	N/A
response to bacterium	N/A	N/A	N/A	N/A
granulocyte chemotaxis	N/A	N/A	N/A	N/A
humoral immune response	N/A	N/A	N/A	N/A
regulation of response to stimulus	N/A	N/A	N/A	N/A
positive regulation of gene expression	N/A	N/A	N/A	N/A
defense response	N/A	N/A	N/A	N/A
positive regulation of protein phosphorylation	N/A	N/A	N/A	N/A
positive regulation of phosphorylation	N/A	N/A	N/A	N/A
positive regulation of cell development	N/A	N/A	N/A	N/A
response to stimulus	N/A	N/A	N/A	N/A
positive regulation of MAPK cascade	N/A	N/A	N/A	N/A
regulation of anatomical structure morphogenesis	N/A	N/A	N/A	N/A
response to lipid	N/A	N/A	N/A	N/A
regulation of chemotaxis	N/A	N/A	N/A	N/A
positive regulation of multicellular organismal process	N/A	N/A	N/A	N/A
positive regulation of lymphocyte activation	N/A	N/A	N/A	N/A
positive regulation of cell adhesion	N/A	N/A	N/A	N/A
positive regulation of biological process	N/A	N/A	N/A	N/A
regulation of protein phosphorylation	N/A	N/A	N/A	N/A
positive regulation of cellular process	N/A	N/A	N/A	N/A
positive regulation of phosphorus metabolic process	N/A	N/A	N/A	N/A
positive regulation of phosphate metabolic process	N/A	N/A	N/A	N/A
regulation of lymphocyte activation	N/A	N/A	N/A	N/A
defense response to symbiont	N/A	N/A	N/A	N/A

positive regulation of leukocyte activation	N/A	N/A	N/A	N/A
regulation of lymphocyte proliferation	N/A	N/A	N/A	N/A
cell population proliferation	N/A	N/A	N/A	N/A
regulation of apoptotic process	N/A	N/A	N/A	N/A
regulation of mononuclear cell proliferation	N/A	N/A	N/A	N/A
positive regulation of cell activation	N/A	N/A	N/A	N/A
positive regulation of chemotaxis	N/A	N/A	N/A	N/A
regulation of phosphorylation	N/A	N/A	N/A	N/A
positive regulation of protein modification process	N/A	N/A	N/A	N/A
regulation of programmed cell death	N/A	N/A	N/A	N/A
response to oxygen-containing compound	N/A	N/A	N/A	N/A
positive regulation of response to external stimulus	N/A	N/A	N/A	N/A
positive regulation of leukocyte migration	N/A	N/A	N/A	N/A
regulation of leukocyte activation	N/A	N/A	N/A	N/A
positive regulation of peptidyl-tyrosine phosphorylation	N/A	N/A	N/A	N/A
regulation of leukocyte proliferation	N/A	N/A	N/A	N/A
regulation of cell adhesion	N/A	N/A	N/A	N/A
response to stress	N/A	N/A	N/A	N/A
positive regulation of developmental process	N/A	N/A	N/A	N/A
regulation of intracellular signal transduction	N/A	N/A	N/A	N/A
regulation of cell activation	N/A	N/A	N/A	N/A
regulation of MAPK cascade	N/A	N/A	N/A	N/A
positive regulation of intracellular signal transduction	N/A	N/A	N/A	N/A
regulation of multicellular organismal development	N/A	N/A	N/A	N/A
positive regulation of cell differentiation	N/A	N/A	N/A	N/A
regulation of phosphate metabolic process	N/A	N/A	N/A	N/A
regulation of phosphorus metabolic process	N/A	N/A	N/A	N/A
regulation of developmental process	N/A	N/A	N/A	N/A
positive regulation of leukocyte differentiation	N/A	N/A	N/A	N/A
positive regulation of hemopoiesis	N/A	N/A	N/A	N/A
negative regulation of biological process	N/A	N/A	N/A	N/A
positive regulation of cell communication	N/A	N/A	N/A	N/A
positive regulation of signaling	N/A	N/A	N/A	N/A
regulation of adaptive immune response based on somatic recombination of immune receptors built from immunoglobulin superfamily domains	N/A	N/A	N/A	N/A
regulation of cell-cell adhesion	N/A	N/A	N/A	N/A
positive regulation of apoptotic process	N/A	N/A	N/A	N/A
regulation of protein modification process	N/A	N/A	N/A	N/A
regulation of peptidyl-tyrosine phosphorylation	N/A	N/A	N/A	N/A
regulation of adaptive immune response	N/A	N/A	N/A	N/A
mononuclear cell migration	N/A	N/A	N/A	N/A
positive regulation of protein metabolic process	N/A	N/A	N/A	N/A
cell activation	N/A	N/A	N/A	N/A
positive regulation of macromolecule biosynthetic process	N/A	N/A	N/A	N/A
mononuclear cell differentiation	N/A	N/A	N/A	N/A
regulation of leukocyte chemotaxis	N/A	N/A	N/A	N/A
positive regulation of signal transduction	N/A	N/A	N/A	N/A
regulation of signal transduction	N/A	N/A	N/A	N/A
positive regulation of cellular biosynthetic process	N/A	N/A	N/A	N/A
regulation of leukocyte cell-cell adhesion	N/A	N/A	N/A	N/A
negative regulation of fat cell differentiation	N/A	N/A	N/A	N/A
regulation of T cell activation	N/A	N/A	N/A	N/A
positive regulation of biosynthetic process	N/A	N/A	N/A	N/A
apoptotic process	N/A	N/A	N/A	N/A
regulation of immune effector process	N/A	N/A	N/A	N/A

regulation of defense response	N/A	N/A	N/A	N/A
positive regulation of cellular metabolic process	N/A	N/A	N/A	N/A
regulation of leukocyte mediated immunity	N/A	N/A	N/A	N/A
positive regulation of production of molecular mediator of immune response	N/A	N/A	N/A	N/A
regulation of cell development	N/A	N/A	N/A	N/A
programmed cell death	N/A	N/A	N/A	N/A
cell death	N/A	N/A	N/A	N/A
leukocyte activation	N/A	N/A	N/A	N/A
cell-cell signaling	N/A	N/A	N/A	N/A
enzyme-linked receptor protein signaling pathway	N/A	N/A	N/A	N/A
leukocyte differentiation	N/A	N/A	N/A	N/A
regulation of biological process	N/A	N/A	N/A	N/A
positive regulation of tyrosine phosphorylation of STAT protein	N/A	N/A	N/A	N/A
regulation of morphogenesis of an epithelium	N/A	N/A	N/A	N/A
homeostatic process	N/A	N/A	N/A	N/A
negative regulation of cellular process	N/A	N/A	N/A	N/A
positive regulation of molecular function	N/A	N/A	N/A	N/A
regulation of protein metabolic process	N/A	N/A	N/A	N/A
positive regulation of immune response	N/A	N/A	N/A	N/A
regulation of tyrosine phosphorylation of STAT protein	N/A	N/A	N/A	N/A
positive regulation of macromolecule metabolic process	N/A	N/A	N/A	N/A
regulation of cellular process	N/A	N/A	N/A	N/A
regulation of signaling	N/A	N/A	N/A	N/A
regulation of cell communication	N/A	N/A	N/A	N/A
regulation of tumor necrosis factor superfamily cytokine production	N/A	N/A	N/A	N/A
anatomical structure morphogenesis	N/A	N/A	N/A	N/A
biological regulation	N/A	N/A	N/A	N/A
positive regulation of cytokine production	N/A	N/A	N/A	N/A
regulation of cell differentiation	N/A	N/A	N/A	N/A
regulation of T cell proliferation	N/A	N/A	N/A	N/A
regulation of leukocyte differentiation	N/A	N/A	N/A	N/A
monocyte chemotaxis	N/A	N/A	N/A	N/A
eosinophil chemotaxis	N/A	N/A	N/A	N/A
negative regulation of immune system process	N/A	N/A	N/A	N/A
positive regulation of cell-cell adhesion	N/A	N/A	N/A	N/A
regulation of molecular function	N/A	N/A	N/A	N/A
eosinophil migration	N/A	N/A	N/A	N/A
regulation of production of molecular mediator of immune response	N/A	N/A	N/A	N/A
regulation of lymphocyte mediated immunity	N/A	N/A	N/A	N/A
positive regulation of leukocyte chemotaxis	N/A	N/A	N/A	N/A
regulation of cytokine production	N/A	N/A	N/A	N/A
positive regulation of metabolic process	N/A	N/A	N/A	N/A
positive regulation of GTPase activity	N/A	N/A	N/A	N/A
negative regulation of multicellular organismal process	N/A	N/A	N/A	N/A
regulation of inflammatory response	N/A	N/A	N/A	N/A
regulation of hemopoiesis	N/A	N/A	N/A	N/A
cell surface receptor protein tyrosine kinase signaling pathway	N/A	N/A	N/A	N/A
positive regulation of T cell activation	N/A	N/A	N/A	N/A
positive regulation of immune effector process	N/A	N/A	N/A	N/A
lymphocyte proliferation	N/A	N/A	N/A	N/A

positive regulation of adaptive immune response based on somatic recombination of immune receptors built from immunoglobulin superfamily domains	N/A	N/A	N/A	N/A
negative regulation of programmed cell death	N/A	N/A	N/A	N/A
mononuclear cell proliferation	N/A	N/A	N/A	N/A
positive regulation of adaptive immune response	N/A	N/A	N/A	N/A
regulation of hydrolase activity	N/A	N/A	N/A	N/A
regulation of natural killer cell chemotaxis	N/A	N/A	N/A	N/A
cellular homeostasis	N/A	N/A	N/A	N/A
positive regulation of lymphocyte differentiation	N/A	N/A	N/A	N/A
regulation of GTPase activity	N/A	N/A	N/A	N/A
positive regulation of leukocyte cell-cell adhesion	N/A	N/A	N/A	N/A
hemopoiesis	N/A	N/A	N/A	N/A
positive regulation of receptor signaling pathway via STAT	N/A	N/A	N/A	N/A
regulation of mononuclear cell migration	N/A	N/A	N/A	N/A
positive regulation of lymphocyte proliferation	N/A	N/A	N/A	N/A
cell differentiation	N/A	N/A	N/A	N/A
cellular developmental process	N/A	N/A	N/A	N/A
positive regulation of mononuclear cell proliferation	N/A	N/A	N/A	N/A
lymphocyte activation	N/A	N/A	N/A	N/A
lymphocyte differentiation	N/A	N/A	N/A	N/A
leukocyte proliferation	N/A	N/A	N/A	N/A
neutrophil chemotaxis	N/A	N/A	N/A	N/A
lymphocyte migration	N/A	N/A	N/A	N/A
positive regulation of leukocyte proliferation	N/A	N/A	N/A	N/A
positive regulation of myeloid leukocyte differentiation	N/A	N/A	N/A	N/A
regulation of tumor necrosis factor production	N/A	N/A	N/A	N/A
regulation of response to stress	N/A	N/A	N/A	N/A
regulation of interleukin-12 production	N/A	N/A	N/A	N/A
neutrophil migration	N/A	N/A	N/A	N/A
regulation of lymphocyte migration	N/A	N/A	N/A	N/A
positive regulation of chemokine production	N/A	N/A	N/A	N/A
regulation of tissue remodeling	N/A	N/A	N/A	N/A
regulation of receptor signaling pathway via STAT	N/A	N/A	N/A	N/A
chemical homeostasis	N/A	N/A	N/A	N/A
positive regulation of macrophage differentiation	N/A	N/A	N/A	N/A
positive regulation of smooth muscle cell proliferation	N/A	N/A	N/A	N/A
regulation of transport	N/A	N/A	N/A	N/A
regulation of animal organ morphogenesis	N/A	N/A	N/A	N/A
calcium ion homeostasis	N/A	N/A	N/A	N/A
positive regulation of mononuclear cell migration	N/A	N/A	N/A	N/A
positive regulation of defense response	N/A	N/A	N/A	N/A
positive regulation of hydrolase activity	N/A	N/A	N/A	N/A
adaptive immune response	N/A	N/A	N/A	N/A
anatomical structure development	N/A	N/A	N/A	N/A
negative regulation of signal transduction	N/A	N/A	N/A	N/A
positive regulation of myeloid cell differentiation	N/A	N/A	N/A	N/A
regulation of macromolecule metabolic process	N/A	N/A	N/A	N/A
regulation of catalytic activity	N/A	N/A	N/A	N/A
regulation of lymphocyte chemotaxis	N/A	N/A	N/A	N/A
regulation of interleukin-1 production	N/A	N/A	N/A	N/A
regulation of ERK1 and ERK2 cascade	N/A	N/A	N/A	N/A
gliogenesis	N/A	N/A	N/A	N/A
positive regulation of cytokine production involved in inflammatory response	N/A	N/A	N/A	N/A

regulation of cytokine production involved in immune response	N/A	N/A	N/A	N/A
regulation of myeloid leukocyte differentiation	N/A	N/A	N/A	N/A
regulation of macromolecule biosynthetic process	N/A	N/A	N/A	N/A
developmental process	N/A	N/A	N/A	N/A
cellular process	N/A	N/A	N/A	N/A
positive regulation of lymphocyte mediated immunity	N/A	N/A	N/A	N/A
regulation of cellular biosynthetic process	N/A	N/A	N/A	N/A
positive regulation of cellular component organization	N/A	N/A	N/A	N/A
regulation of endothelial cell proliferation	N/A	N/A	N/A	N/A
T cell migration	N/A	N/A	N/A	N/A
regulation of biosynthetic process	N/A	N/A	N/A	N/A
positive regulation of catalytic activity	N/A	N/A	N/A	N/A
regulation of smooth muscle cell proliferation	N/A	N/A	N/A	N/A
positive regulation of leukocyte mediated immunity	N/A	N/A	N/A	N/A
myeloid leukocyte activation	N/A	N/A	N/A	N/A
myeloid leukocyte differentiation	N/A	N/A	N/A	N/A
regulation of metabolic process	N/A	N/A	N/A	N/A
angiogenesis	N/A	N/A	N/A	N/A
regulation of localization	N/A	N/A	N/A	N/A
regulation of cellular component organization	N/A	N/A	N/A	N/A
regulation of cellular metabolic process	N/A	N/A	N/A	N/A
tube development	N/A	N/A	N/A	N/A

Supplementary table 6. Reactome overrepresentation analysis (https://reactome.org/PathwayBrowser/#TOOL=AT) was performed to the up-regulated target (30 analytes) and background proteins (49 analytes) and the specific biological processes were identified using InteractiVenn (https://www.interactivenn.net/). Enriched processes are listed according to their P-values (P<0.01). Note that the enrichment values are not not given for the target and background comparison, due to the fact that these processes have two separate values from the analyses. FDR=False discovery rate. N/A=Data not available.

Overrepresented processes identified with the target set of proteins (P<0.01)				
Pathway	Number of proteins	Total protein number	P value	FDR
NFE2L2 regulating inflammation associated genes	3	7	1.5E-6	5.11E-5
ATF4 activates genes in response to endoplasmic reticulum stress	4	34	3.94E-6	1.03E-4
PERK regulates gene expression	4	42	1E-5	2.16E-4
Senescence-Associated Secretory Phenotype (SASP)	4	90	1.71E-4	3.76E-3
MAPK family signaling cascades	6	367	8.57E-4	1.14E-2
FGFRL1 modulation of FGFR1 signaling	2	14	8.7E-4	1.14E-2
CD163 mediating an anti-inflammatory response	2	14	8.7E-4	1.14E-2
Platelet degranulation	4	142	9.43E-4	1.14E-2
Signaling by activated point mutants of FGFR1	2	15	9.96E-4	1.14E-2
Regulation of gene expression by Hypoxia-inducible Factor	2	15	9.96E-4	1.14E-2
Response to elevated platelet cytosolic Ca2+	4	149	1.13E-3	1.14E-2
FGFR2c ligand binding and activation	2	17	1.27E-3	1.14E-2
FGFR1c ligand binding and activation	2	17	1.27E-3	1.14E-2
Signaling by activated point mutants of FGFR3	2	17	1.27E-3	1.14E-2
FGFR3 mutant receptor activation	2	17	1.27E-3	1.14E-2
Unfolded Protein Response (UPR)	4	156	1.33E-3	1.14E-2
FGFR3c ligand binding and activation	2	18	1.43E-3	1.14E-2
Phospholipase C-mediated cascade; FGFR3	2	18	1.43E-3	1.14E-2
FGFR3 ligand binding and activation	2	19	1.59E-3	1.27E-2
STAT3 nuclear events downstream of ALK signaling	2	19	1.59E-3	1.27E-2
Phospholipase C-mediated cascade: FGFR1	2	22	2.11E-3	1.47E-2
FGFR1 ligand binding and activation	2	23	2.31E-3	1.47E-2
Activated point mutants of FGFR2	2	23	2.31E-3	1.47E-2
PI-3K cascade:FGFR3	2	24	2.51E-3	1.47E-2
Phospholipase C-mediated cascade; FGFR2	2	25	2.71E-3	1.47E-2
MAPK1/MAPK3 signaling	5	316	2.74E-3	1.47E-2
Leishmania parasite growth and survival	4	192	2.82E-3	1.47E-2
Anti-inflammatory response favouring Leishmania parasite infection	4	192	2.82E-3	1.47E-2
SHC-mediated cascade:FGFR3	2	26	2.93E-3	1.47E-2
FGFR2 ligand binding and activation	2	26	2.93E-3	1.47E-2
Signaling by Receptor Tyrosine Kinases	7	634	2.99E-3	1.49E-2
FRS-mediated FGFR3 signaling	2	27	3.15E-3	1.55E-2
Cellular Senescence	4	199	3.2E-3	1.55E-2
PI-3K cascade:FGFR1	2	28	3.39E-3	1.55E-2
Cellular responses to stress	9	1,040	3.85E-3	1.55E-2

SHC-mediated cascade:FGFR1	2	30	3.87E-3	1.55E-2
Interleukin-6 family signaling	2	30	3.87E-3	1.55E-2
Assembly of active LPL and LIPC lipase complexes	2	30	3.87E-3	1.55E-2
Constitutive Signaling by Aberrant PI3K in Cancer	3	104	4.01E-3	1.6E-2
FRS-mediated FGFR1 signaling	2	31	4.13E-3	1.65E-2
PI-3K cascade:FGFR2	2	31	4.13E-3	1.65E-2
SHC-mediated cascade:FGFR2	2	33	4.66E-3	1.83E-2
Signaling by FGFR3 in disease	2	33	4.66E-3	1.83E-2
FRS-mediated FGFR2 signaling	2	34	4.93E-3	1.83E-2
Negative regulation of FGFR3 signaling	2	34	4.93E-3	1.83E-2
Downstream signaling of activated FGFR3	2	35	5.22E-3	1.83E-2
Molecules associated with elastic fibres	2	37	5.81E-3	1.83E-2
TGFBR2 MSI Frameshift Mutants in Cancer	1	2	6.08E-3	1.83E-2
Drug-mediated inhibition of MET activation	1	2	6.08E-3	1.83E-2
Negative regulation of FGFR1 signaling	2	39	6.43E-3	1.93E-2
FGFR1 mutant receptor activation	2	39	6.43E-3	1.93E-2
Negative regulation of FGFR2 signaling	2	41	7.08E-3	2.12E-2
Downstream signaling of activated FGFR1	2	42	7.41E-3	2.22E-2
Downstream signaling of activated FGFR2	2	42	7.41E-3	2.22E-2
PI3K/AKT Signaling in Cancer	3	132	7.72E-3	2.23E-2
FGFR2 mutant receptor activation	2	43	7.75E-3	2.23E-2
Cellular responses to stimuli	9	1,167	8.11E-3	2.23E-2
Nuclear events mediated by NFE2L2	3	135	8.2E-3	2.23E-2
Purine salvage	2	45	8.46E-3	2.23E-2
Elastic fibre formation	2	45	8.46E-3	2.23E-2
PI5P, PP2A and IER3 Regulate PI3K/AKT Signaling	3	137	8.54E-3	2.23E-2
Signaling by ALK	2	46	8.82E-3	2.23E-2
Transcriptional Regulation by VENTX	2	48	9.57E-3	2.23E-2
Negative regulation of the PI3K/AKT network	3	145	9.95E-3	2.23E-2
Signaling by FGFR1 in disease	2	49	9.95E-3	2.23E-2
Overrepresented processes identified with the background set of proteins (P<0.01)				
Pathway	Number of proteins	Fold enrichment	Raw P value	FDR
Interleukin-18 signaling	4	11	2.06E-7	5.77E-6
Signaling by GPCR	14	878	2.65E-5	5.57E-4
Interleukin-1 family signaling	6	170	1.12E-4	2.01E-3
GPCR downstream signalling	12	795	1.8E-4	2.88E-3
TNFs bind their physiological receptors	3	30	3.39E-4	4.74E-3
RUNX1 regulates transcription of genes involved in interleukin signaling	2	7	4.63E-4	6.48E-3
TRAIL signaling	2	8	6.03E-4	7.23E-3
TNFR2 non-canonical NF-kB pathway	4	91	7.4E-4	8.37E-3

RUNX1 regulates transcription of genes involved in WNT signaling	2	9	7.61E-4	8.37E-3
RUNX1 regulates estrogen receptor mediated transcription	2	10	9.36E-4	1.03E-2
Regulation by c-FLIP	2	11	1.13E-3	1.13E-2
Dimerization of procaspase-8	2	11	1.13E-3	1.13E-2
CASP8 activity is inhibited	2	12	1.34E-3	1.21E-2
Signaling by AXIN mutants	2	15	2.08E-3	1.87E-2
Regulation of Insulin-like Growth Factor (IGF) transport and uptake by Insulin-like Growth Factor Binding Proteins (IGFBPs)	4	127	2.49E-3	2.12E-2
TNF receptor superfamily (TNFSF) members mediating non-canonical NF-kB pathway	2	17	2.65E-3	2.12E-2
Caspase activation via Death Receptors in the presence of ligand	2	20	3.64E-3	2.8E-2
Collagen degradation	3	69	3.65E-3	2.8E-2
Deletions in the AXIN1 gene destabilize the destruction complex	1	1	4.41E-3	3.09E-2
Regulated Necrosis	3	76	4.77E-3	3.18E-2
Estrogen-dependent gene expression	4	153	4.82E-3	3.18E-2
Other interleukin signaling	2	24	5.18E-3	3.18E-2
Transcriptional and post-translational regulation of MITF-M expression and activity	3	79	5.3E-3	3.18E-2
ESR-mediated signaling	5	257	5.7E-3	3.42E-2
TNFR1-induced proapoptotic signaling	2	26	6.05E-3	3.63E-2
Formation of the ureteric bud	2	29	7.46E-3	4.48E-2
Caspase activation via extrinsic apoptotic signalling pathway	2	32	9.01E-3	5.4E-2
Diseases of signal transduction by growth factor receptors and second messengers	7	536	9.51E-3	5.59E-2
Overrepresented processes identified with both the target set and the background set of proteins (P<0.01)				
Pathway				
Interleukin-10 signaling	N/A	N/A	N/A	N/A
Signaling by Interleukins	N/A	N/A	N/A	N/A
Cytokine Signaling in Immune system	N/A	N/A	N/A	N/A
Interleukin-4 and Interleukin-13 signaling	N/A	N/A	N/A	N/A
Immune System	N/A	N/A	N/A	N/A
Chemokine receptors bind chemokines	N/A	N/A	N/A	N/A
Peptide ligand-binding receptors	N/A	N/A	N/A	N/A
Class A/1 (Rhodopsin-like receptors)	N/A	N/A	N/A	N/A
Signal Transduction	N/A	N/A	N/A	N/A
TFAP2 (AP-2) family regulates transcription of growth factors and their receptors	N/A	N/A	N/A	N/A
GPCR ligand binding	N/A	N/A	N/A	N/A
Interleukin-20 family signaling	N/A	N/A	N/A	N/A
Activation of Matrix Metalloproteinases	N/A	N/A	N/A	N/A
G alpha (i) signalling events	N/A	N/A	N/A	N/A

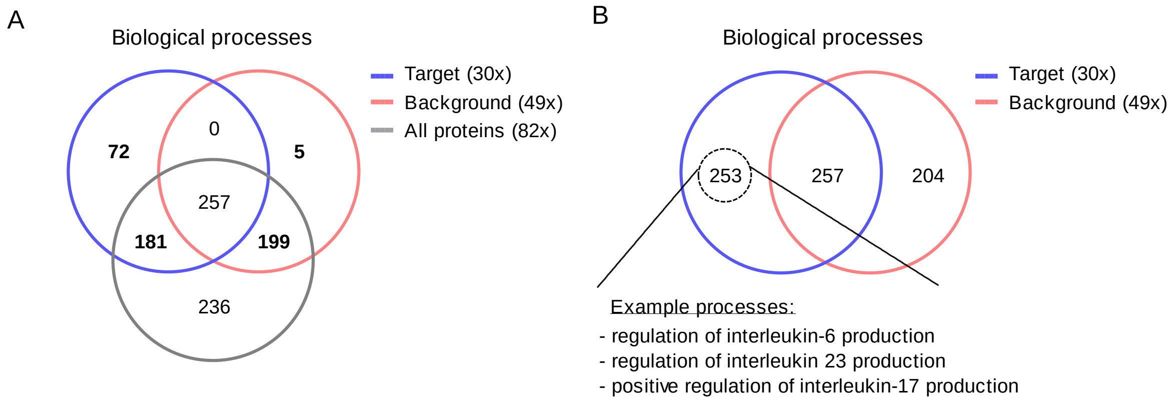

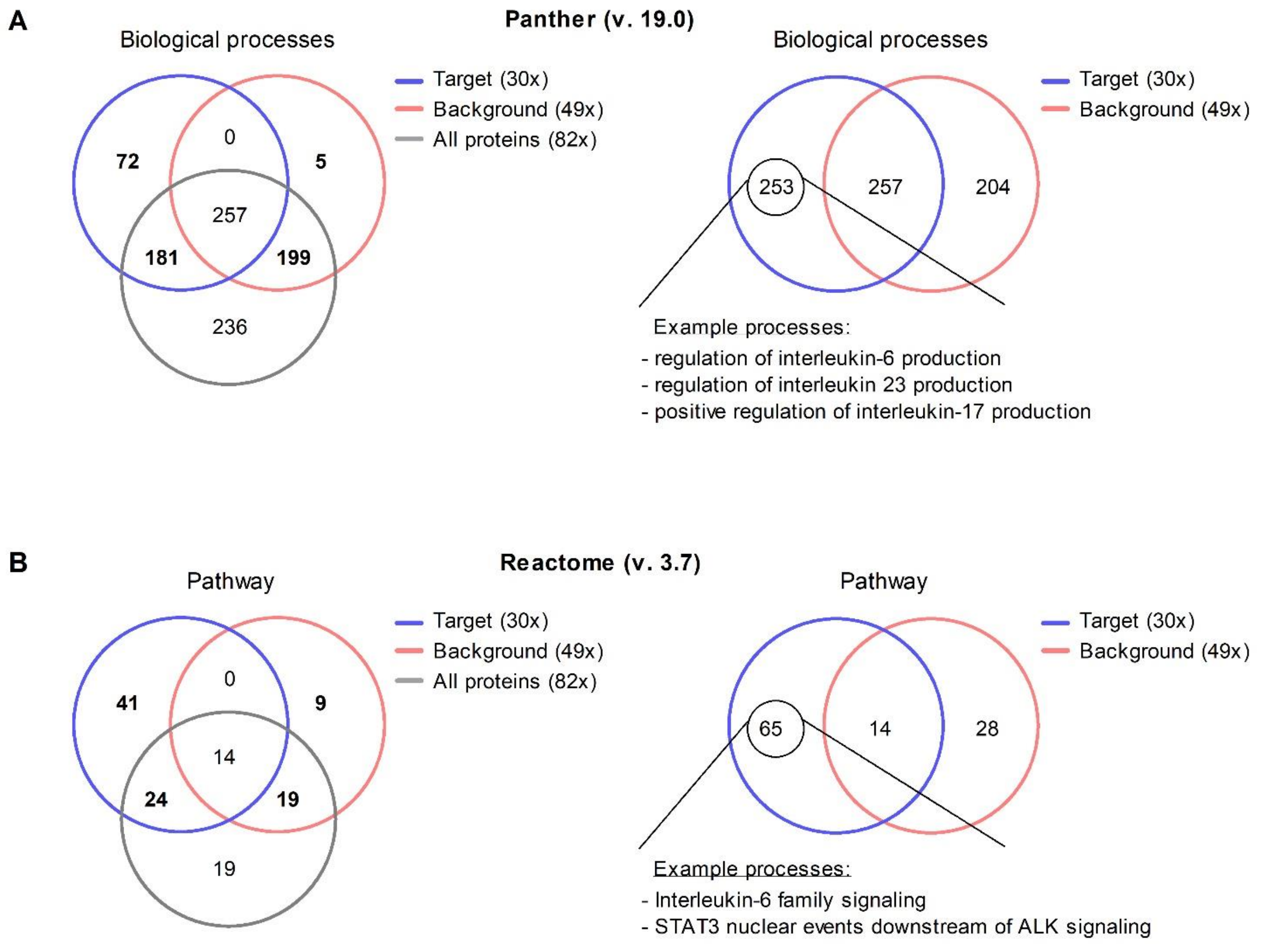

Supplementary Figure 1. Comparison of overrepresented biological processes in upregulated (target) and similarly expressed (background) protein sets between cases and controls. Overrepresentation analysis was performed using **A)** Panther (v. 13.0) or **B)** Reactome (v. 3.7) on the target proteins (30 analytes), background proteins (49 analytes), and all measured proteins (82 analytes). The identified biological processes are shown as Venn diagrams. Notably, downregulated proteins (3 analytes) did not reveal any overrepresented processes and are therefore excluded from the figure. Left panels show the comparison of enriched processes across all three protein sets, while right panels show the specific identification of overrepresented biological processes

between the target and background groups, including examples of target-specific processes related to interleukin (IL) signaling.
